# Integration of multi-source gene interaction networks and omics data with graph attention networks to identify novel disease genes

**DOI:** 10.1093/bioinformatics/btaf181

**Published:** 2025-04-23

**Authors:** Kaiyuan Yang, Jiabei Cheng, Shenghao Cao, Xiaoyong Pan, Hong-bin Shen, Jin Cheng, Ye Yuan

**Affiliations:** Key Laboratory of Biopharmaceutical Preparation and Delivery, State Key Laboratory of Biochemical Engineering, Institute of Process Engineering, Chinese Academy of Sciences, Beijing 100190, China; Institute of Image Processing and Pattern Recognition, Shanghai Jiao Tong University, Shanghai 200240, China; Key Laboratory of System Control and Information Processing, Ministry of Education of China, Shanghai 200240, China; Institute of Image Processing and Pattern Recognition, Shanghai Jiao Tong University, Shanghai 200240, China; Key Laboratory of System Control and Information Processing, Ministry of Education of China, Shanghai 200240, China; Institute of Image Processing and Pattern Recognition, Shanghai Jiao Tong University, Shanghai 200240, China; Key Laboratory of System Control and Information Processing, Ministry of Education of China, Shanghai 200240, China; Institute of Image Processing and Pattern Recognition, Shanghai Jiao Tong University, Shanghai 200240, China; Key Laboratory of System Control and Information Processing, Ministry of Education of China, Shanghai 200240, China; Institute of Image Processing and Pattern Recognition, Shanghai Jiao Tong University, Shanghai 200240, China; Key Laboratory of System Control and Information Processing, Ministry of Education of China, Shanghai 200240, China; Medical Robot Research Institute, School of Biomedical Engineering, Shanghai Jiao Tong University, Shanghai 200240, China; Key Laboratory of Biopharmaceutical Preparation and Delivery, State Key Laboratory of Biochemical Engineering, Institute of Process Engineering, Chinese Academy of Sciences, Beijing 100190, China

## Abstract

**Motivation:**

The pathogenesis of diseases is closely associated with genes, and the discovery of disease genes holds significant importance for understanding disease mechanisms and designing targeted therapeutics. However, biological validation of all genes for diseases is expensive and challenging.

**Results:**

In this study, we propose DGP-AMIO, a computational method based on graph attention networks, to rank all unknown genes and identify potential novel disease genes by integrating multi-omics and gene interaction networks from multiple data sources. DGP-AMIO outperforms other methods significantly on 20 disease datasets, with an average AUROC and AUPR exceeding 0.9. The superior performance of DGP-AMIO is attributed to the integration of multiomics and gene interaction networks from multiple databases, as well as triGAT, a proposed GAT-based method that enables precise identification of disease genes in directed gene networks. Enrichment analysis conducted on the top 100 genes predicted by DGP-AMIO and literature research revealed that a majority of enriched GO terms, KEGG pathways and top genes were associated with diseases supported by relevant studies. We believe that our method can serve as an effective tool for identifying disease genes and guiding subsequent experimental validation efforts.

**Availability and implementation:**

DGP-AMIO is publicly available at https://github.com/yangkaiyuan1027/DGP-AMIO.

## 1 Introduction

Researches have indicated a close association between diseases and genes (Jackson *et al.* 2018, Zheng *et al.* 2019), and the existing catalog of disease genes is considered incomplete. Therefore, the discovery of disease genes holds profound significance as it contributes to our understanding of disease mechanisms and facilitates the design of targeted therapeutics (Linehan *et al.* 2007). However, the wet-lab experiments required to validate gene–disease associations are time-consuming and expensive. Moreover, considering the vast number of human genes, it is unfeasible to perform biological experiments for all genes. Hence, accurate and efficient computational tools need to be developed to prioritize candidate genes and select those most likely to be associated with diseases. This would guide subsequent experimental validation and enhance the overall efficiency of the entire process.

The role of genes in diseases is diverse and complex. Some genes undergo mutations directly associated with diseases (Machackova *et al.* 2008), while others contribute to disease occurrence through abnormal expression (Zheng *et al.* 2019). Additionally, research has shown an association between DNA methylation and diseases (Calle-Fabregat *et al.* 2020). Moreover, genes interact in intricate ways, including signal transduction, regulatory pathways, and protein–protein interactions (PPIs). Some genes exert their effects on diseases by regulating or interacting with other genes (Hu *et al.* 2019, Zhang *et al.* 2019). Inspired by these insights, numerous methods have been proposed for identifying disease genes. One category is based on epigenetics, where machine learning models are trained using omics data such as gene expression data to identify disease genes (Xiao *et al.* 2011, Yuan and Bar-Joseph 2019). Another category involves constructing gene interaction networks, such as PPI networks, and using traditional graph machine learning methods to extract topological features of nodes to identify disease genes (Liu *et al.* 2020, Zhang *et al.* 2021). These aforementioned methods only utilize a single type of data, either multidimensional omics data or gene interactions. Considering the complexity of the gene–disease associations, integrating multiple types of data becomes necessary. The advancement of graph neural network (GNN) techniques provides a viable framework for this integration, where genes serve as nodes, gene interactions as edges, and multidimensional omics data as node features. This led to the emergence of GNN-based methods (Schulte-Sasse *et al.* 2021, Azadifar and Ahmadi 2022, Zhang *et al.* 2022). EMOGI (Schulte-Sasse *et al.* 2021), for instance, combines PPI networks with four types of omics data using graph convolutional network (GCN) (Kipf and Welling 2017) to identify cancer genes, demonstrating the significant advantages of integrating gene interaction networks and omics data over using a single data type alone.

Nevertheless, there are some limitations in existing graph neural network-based methods. Firstly, the majority of these methods construct undirected graphs based on PPI networks. However, gene regulations and signaling pathways are directional, which are valuable sources of knowledge for predicting disease genes. Furthermore, there are diverse types of gene interaction networks available, including PPI networks, gene regulatory networks, and KEGG (Kanehisa and Goto 2000) pathways, each originating from various databases. Existing methods often require training on separate networks from different databases. However, the performance of these models exhibits considerable variations across different databases, leading to disparate predictions for the same gene. Consequently, it is necessary to integrate gene interaction networks from multiple databases, constructing a more comprehensive knowledge graph and enabling more precise predictions of disease genes.

To address the issues mentioned above, we proposed a **D**isease **G**ene **P**redictor based on **A**ttention mechanism and integration of **M**ulti-source gene **I**nteraction networks and **O**mics (DGP-AMIO). We merged gene interaction networks of different types and databases into a unified directed graph. Moreover, we introduced a 0/1 vector on the edges to indicate the presence or absence of gene interactions in each database and incorporated this edge feature into the training of attention coefficients. Additionally, we proposed triGAT improved based on graph attention networks (Veličković *et al.* 2017) (GAT), which better utilizes the relationships between genes and their upstream/downstream genes for disease gene prediction. Experimental results demonstrate that DGP-AMIO outperforms other methods on multiple diseases, achieving an average AUROC and AUPR above 0.9 on the test set. Ablation experiments show that both improvement strategies have a positive impact on the model performance. Furthermore, gene enrichment analysis and literature review provide ample support evidence for the predictions made by DGP-AMIO. Overall, DGP-AMIO is a general computational tool capable of accurately predicting novel disease genes. The predictions by DGP-AMIO have valuable implications for guiding subsequent experimental validations.

## 2 Materials and methods

### 2.1 Data acquisition and preprocessing

#### 2.1.1 Gene expression data

We mainly collected gene expression data from GEO, and for cancer gene prediction, TCGA is also an available database providing different types of omics data. We averaged duplicate gene expression and deleted genes whose expression quantities of all samples are zero. Expression data only is enough for model training, we can use other types of omics such as DNA methylation or gene mutation and concatenate them all for better performance.

#### 2.1.2 Integration of multiple gene interaction networks

We collected gene regulatory networks including KEGG (Kanehisa and Goto 2000), RegNetwork (Liu *et al.* 2015), TRRUST (Han *et al.* 2015), EVEX (Van Landeghem *et al.* 2012), JASPAR (Mathelier *et al.* 2014), CHEA (Lachmann *et al.* 2010), MOTIFMAP (Xie *et al.* 2009), and PPI networks including CPDB (Kamburov *et al.* 2013), STRING (Szklarczyk *et al.* 2019), and IRefIndex (Razick *et al.* 2008) for network integration. Depending on the networks, we preprocessed the data with different methods. For KEGG, we downloaded 345 pathways of human through KEGGREST, converted them into directed graphs and merged them through KEGGgraph in R, generating a directed graph including all pathways. For CPDB, STRING, and RegNetwork, which contain confidence information of gene–gene interactions, we retained interactions with high confidence. Referring to the processing method in the work of EMOGI (Schulte-Sasse *et al.* 2021), we retained interactions with a score >0.5 for CPDB and >0.85 for STRING, and for RegNetwork we retained gene regulations whose confidence is high or middle. For the IRefIndex, we only retained binary interactions. Networks from other databases can be directly downloaded without further preprocessing.

Before the integration of multiple databases, considering the PPI networks are undirected graph while our model deals with directed graph, we converted each undirected edge in PPI networks into two reverse directed edges. Next, for each network, we deleted duplicated edges and self-loops. After the preprocessing steps described above, each gene network from a database is represented in the form of a *n* × 2 edge list, where n is the number of edges (gene interactions). In directed graph, the first column of the edge list is the source nodes (genes) of directed edges, and the second column is the target nodes of directed edges.

Then we took the union of edges in all databases. For edge i→j, we used a *d*-dimensional vector $e_{ij}=[x_{ij}^{1},x_{ij}^{2},\ldots ,x_{ij}^{k},\ldots ,x_{ij}^{d}]$ composed of 0 and 1 to represent its database information, where d equals the number of databases and $x_{ij}^{k}=1$ if edge *i*→*j* is included in the kth database else $x_{ij}^{k}=0$ (Supplementary Fig. S10 explains this process intuitively). In the model this vector appears as edge feature and is involved in training the attention weights.

#### 2.1.3 Disease genes

We collected known disease genes from Malacards (Rappaport *et al.* 2017), an integrated database of human maladies. These genes are positive samples with label 1 in model training.

#### 2.1.4 Data overlap

For genes without expression (or other omics) data, we deleted the corresponding nodes in the network and their connected edges. Moreover, we removed the known disease genes not covered in the network. In this way we constructed a knowledge graph, where nodes and edges are genes and interactions, gene expression data are node feature, database information is edge feature, and genes with label 1 are disease genes.

### 2.2 Methods

#### 2.2.1 Graph attention networks

Our model structure is based on the graph attention networks (GAT) (Veličković *et al.* 2017). GAT is a spatial GCN based on attention mechanism. During message passing, instead of simply summing or averaging neighbor node features, GAT assigns different importance to neighbor nodes through computing attention coefficient:


(1) αij=exp(LeakyReLU(aT[Wxi||Wxj]))∑k∈Ni∪{i} exp (LeakyReLU(aT[Wxi||Wxk]))

The attention coefficient $\alpha_{ij}$ represents the importance of node j to node i, where $x_{i},x_{j}\in R^{F}$ are node feature, $W\in R^{F^{\prime }\times F}$ is a trainable weight matrix, $a\in R^{{2F}^{\prime }}$ is a weight vector representing a shared single-layer feedforward neural network, and $N_{i}$ is the set of neighbor nodes of node i. In directed graph where information flows in the direction of edges, $N_{i}$ is the set of neighbor nodes pointing to i. Then the model aggregates message from neighbor nodes by computing weighted sum and applying a nonlinearity σ, hence generates the updated representation of node i:


(2) xi′=σ(∑j∈Ni∪{i}αijWxj)

Moreover, GAT applies multi-head attention mechanism, allowing that different attention coefficients are computed between the same nodes by learning $a^{k}$(the kth attention mechanism represented by a single-layer FFN, k=1,…,K), so that GAT can capture richer information. For K-head attention, the output node representation of one GAT layer is computed by concatenating or averaging the K updated node features:


(3) xi′=∥k=1Kσ(∑j∈Ni∪{i}αijkWkxj′) or xi′=σ(1K∑k=1K∑j∈Ni∪{i}αijkWkxj′)

where $\alpha_{ij}^{k}$ is the attention coefficient computed by $a^{k}$, and $W^{k}$ is the corresponding linear transformation weight matrix, both of which are trainable.

#### 2.2.2 triGAT

##### 2.2.2.1 Attention mechanism with edge feature

Considering our model includes edge features which represent the database information, we added them into the calculation of attention coefficients:


(4) αij= exp (LeakyReLU(aT[Wxi||Wxj||Weeij]))∑k∈Ni∪{i} exp (LeakyReLU(aT[Wxi||Wxk||Weeik]))

where $e_{ij}$ is the feature of edge j→i, $W_{e}$ is the transformation weight matrix of edge feature. This allows GAT to automatically learn the importance of different databases.

##### 2.2.2.2 triGAT with message feedback

When GAT handles directed graphs, the message can only flows from the source node to the target node, while cannot be fedback from downstream to upstream. One feasible solution is to convert all directed edges to undirected ones, while this may cause the loss of directional information, which is critical in gene regulatory networks and pathways. To address this problem, we proposed triGAT, which not only retains the directional information in the original digraph, but also enables message feedback from downstream to upstream. One triGAT layer is composed of three single-layer GATs whose input graphs are $G_{1}$, $G_{2}$, and $G_{3}$. $G_{1}$ is the original digraph, $G_{2}$ is the reverse digraph where direction of each edge in $G_{2}$ is opposite to $G_{1}$, and $G_{3}$ is the bidirectional graph where between each connected node pair exists two opposite edges. The input node and edge features of these three GATs are the same. triGAT layer concatenates the learned node representations of three GATs and propagates them forward to the next layer, as shown in Equation (5), where X, E are the input node and edge feature and $X^{\prime }$ is the output node representation matrix of one triGAT layer.


(5) X′=GAT1(X,E,G1)||GAT2(X,E,G2)||GAT3(X,E,G3)

### 2.3 Model training

In disease gene prediction tasks, known disease targets (recorded in Malacards) are positive samples while the other gene labels are unknown, which means we lack samples with clear negative label. Considering minimal genes are related to human diseases, we randomly sampled genes equal to the number of positive samples among the unlabeled genes as negative samples.

After sampling, the labeled data were first randomly split into the training (80%) and test (20%) set, and then the training set was divided into five equal parts for cross validation, with each split keeping the ratio of positive and negative samples unchanged. During training, the training set was used to train five ensembles, each ensemble holding out one part for validation and early stopping. The input of the model was composed of a node feature matrix X, an integrated gene interaction network represented by its adjacency matrix A and edge feature matrix E, and labels y. We applied cross-entropy as loss function [Equation (6), where y is the label and h is the model output after the sigmoid function] for training genes and used ADAM as optimizer to train our model. The early stopping strategy was used to stop training when the loss on validation set failed to decrease for p consecutive epochs.


(6) L=-ylog(h)-(1-y)log⁡(1-h)

### 2.4 Evaluation and prediction

The average output of five ensemble models generated by cross validation was used for calculation of AUROC and AUPR on test set. To further evaluate the precision and generalization, we collected disease-associated genes from DisGeNet (Piñero *et al.* 2017) and AllerGAtlas (Liu *et al.* 2018) and removed the genes that appeared in the training and test set. Then we used the remaining genes as positive and sampled unknown genes as negative to calculate metrics. After evaluation, the model can predict on the whole graph, giving a disease-related probability value for each gene except for known disease genes. We sorted these genes in descending order of the probability and the top genes are more likely to be novel disease targets.

## 3 Results

**Figure 1.**
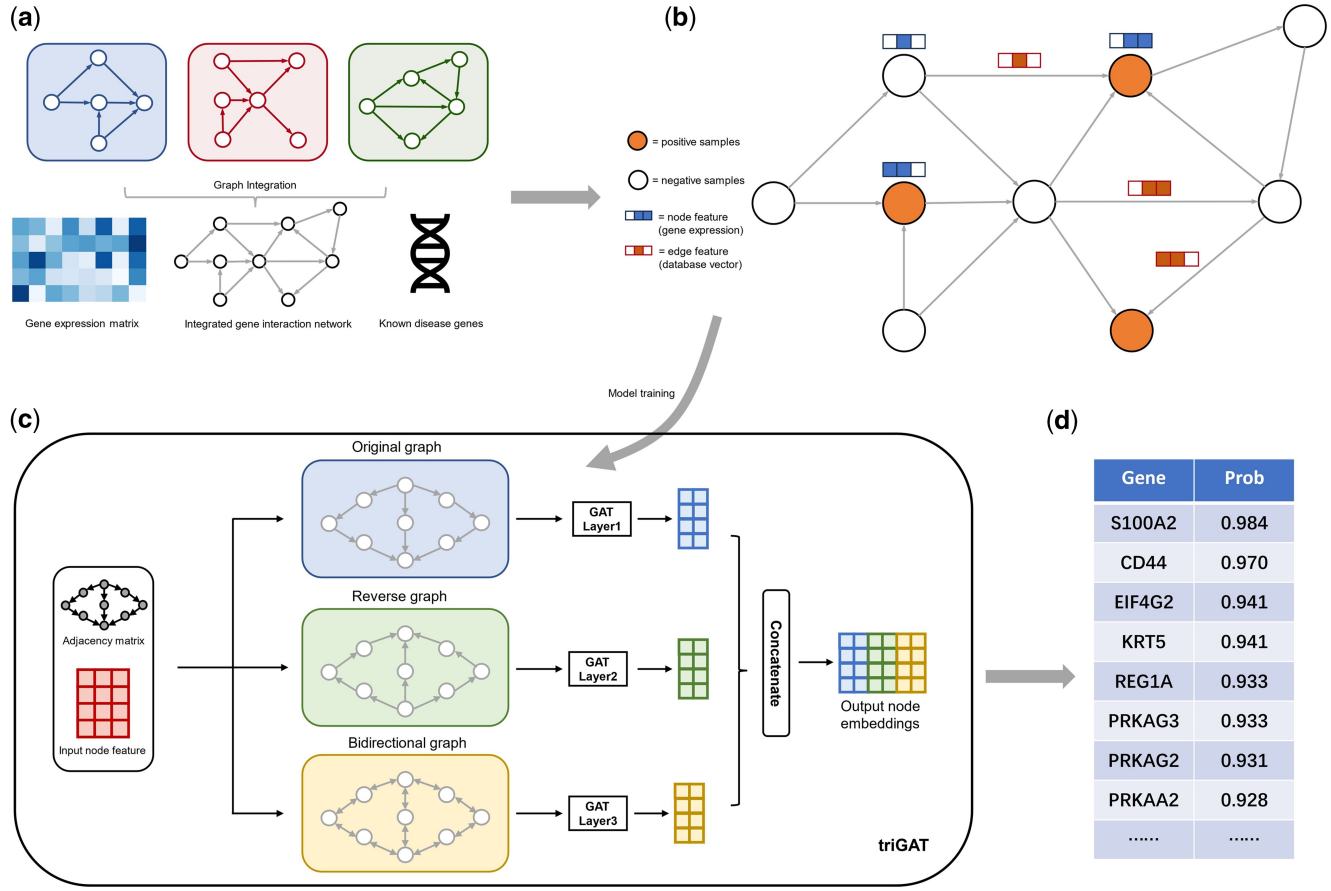
Schematic of DGP-AMIO framework. (a) Data preparation and integration. Gene interaction networks from different databases are integrated to form a unified digraph which is then combined with gene expression (or other omics) data and knowledge of known disease genes. (b) The knowledge graph for training, where nodes correspond to genes, edges to interactions, node features to multidimensional gene expression vectors and edge features to the records of gene interactions in different databases, which are *d*-dimension 0/1 vectors (*d* is the number of databases). (c) Framework of triGAT, proposed in DGP-AMIO to better extract node embeddings in directed graph for node classification. triGAT takes as input the digraphs and is trained on the original graph, reverse graph and bidirectional graph and concatenated the node embeddings of the three graphs to obtain the output. (d) The output of DGP-AMIO, a gene list ranked according to the probability of being related to diseases.

DGP-AMIO is based on GATs and multi-graph integration, and trained in a semi-supervised framework to identify potential disease genes. It constructs a graph from gene interactions of multiple databases and gene expression profiles (Fig. 1a). In detail, gene regulatory networks and PPIs were downloaded from publicly available databases and converted into digraphs respectively, where nodes are genes and edges are interactions between them, and then all nodes and directed edges were taken union. A 0/1 vector was added on each edge to represent the database information, where 1 indicates that the gene interaction was recorded in the database of the vector's corresponding dimension and 0 indicates unrecorded. Preprocessed gene expression or other omics data was combined with the graph as node feature. The graph was partially labeled, where positive samples are known disease genes recorded in Malacards and negative samples are genes randomly sampled from the remaining genes (Fig. 1b). To fully extract the upstream and downstream features of genes, we developed triGAT (Fig. 1c), a GAT-based model which can leverage direction information of edges in the digraph and learn better node representations. The graph was fed into triGAT, and the model was trained on 5-fold cross validation dataset and tested on labeled data that did not appear in the training and validating process. The output of DGP-AMIO is the probabilities of being disease genes on all unlabeled data in the graph (Fig. 1d).

### 3.1 DGP-AMIO precisely identifies disease genes

We trained DGP-AMIO on disease genes from Malacards based on expression data from Gene Expression Omnibus (GEO) and the integration of ten gene interaction networks from publicly available databases. We evaluated DGP-AMIO's performance across different diseases.

#### 3.1.1 Comparison with other methods

We compared DGP-AMIO with other methods for disease gene prediction on 20 diseases, including cancer and non-cancer diseases. For each disease, we divided the dataset into the training and test set and conducted 5-fold cross validation on the training set, and computed the AUROC and AUPR on the test set for each method. Moreover, considering the negative samples are randomly sampled from the unlabeled genes, to reduce the bias introduced in the sampling process, for each disease we sampled five subsets of negative samples using different random seeds. Then we trained and evaluated models separately on these five labeled datasets. To demonstrate the superiority of our method, we benchmarked DGP-AMIO against three methods. CNNC (Yuan and Bar-Joseph 2019) is a deep learning method for inferring gene relationships from expression data based on CNN, which converted the co-expression data of gene pairs into images and fed them into CNN. It can predict new disease genes through their co-expression with known disease genes. EMOGI (Schulte-Sasse *et al.* 2021) is a pan-cancer gene identification method based on GCN, using multi-omics data and PPI networks as input, which can also be used for disease gene prediction. Furthermore, we benchmarked DGP-AMIO against node2vec+RandomForest, which also combines omics data and graph structure information. This method was shown to have similar performance to EMOGI. The graph features learned by node2vec was concatenated with gene expression data after PCA dimension reduction and then were fed into a random forest binary classifier. The dimension of node embeddings learned by node2vec was set to be 32 after optimization experiments (Supplementary Table S3). For fair comparison, we used the same gene expression data for all methods and used STRING PPI network for EMOGI and node2vec+RandomForest.

**Figure 2.**
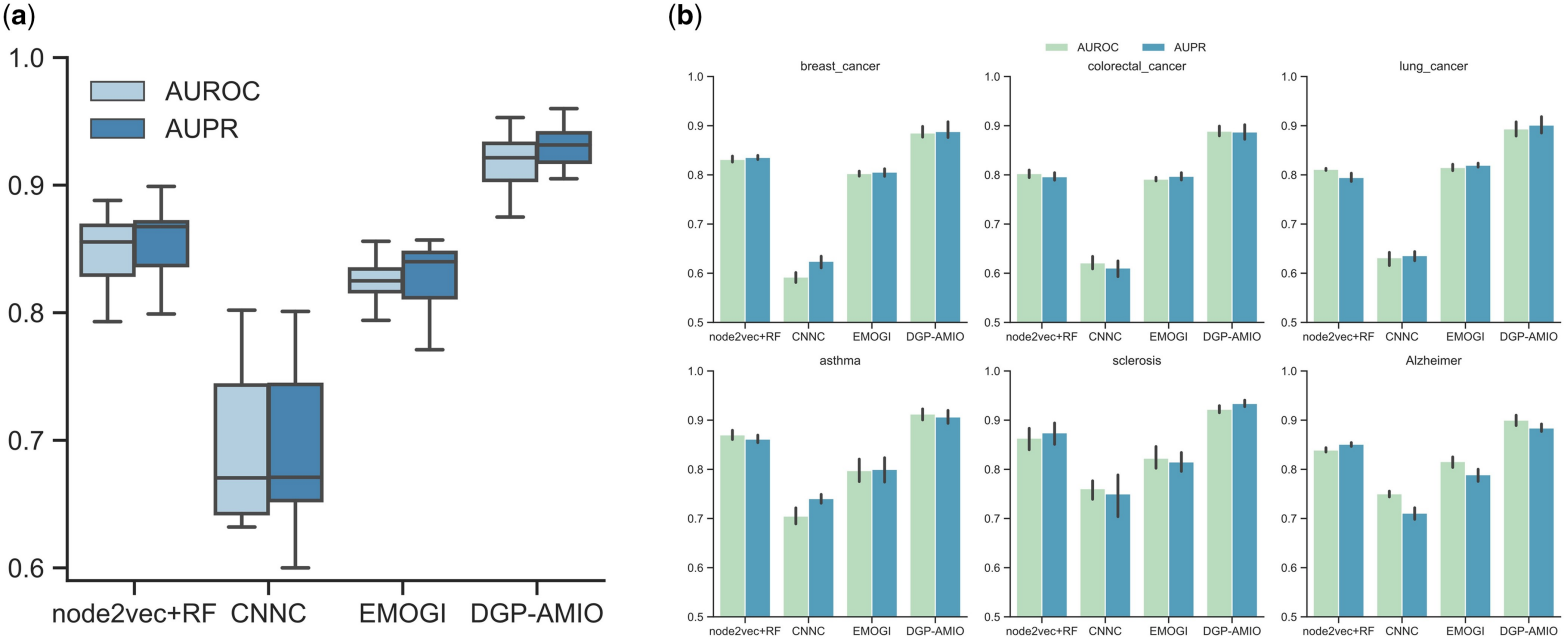
DGP-AMIO outperforms previous methods in predicting disease genes on multiple diseases. (a) AUROCs and AUPRs of different methods, computed on the test sets of 20 diseases. (b) Performances of different methods on six diseases: breast cancer, colorectal cancer, lung cancer, asthma, sclerosis, and Alzheimer. Each method is trained five times on each disease, using the same positive samples and different subsets of negative samples.

The results in Fig. 2a show that the performance of DGP-AMIO significantly outperforms other methods across all diseases, with AUROC and AUPR above 0.9 on the test set for most diseases. Figure 2b shows the performances of models trained with the same positive samples and five different subsets of negative samples on six diseases, showing DGP-AMIO can identify disease genes more precisely than other methods. Results of more diseases are included in Supplementary Fig. S3.

#### 3.1.2 triGAT improves DGP-AMIO’s capability to identify both upstream and downstream disease genes

**Figure 3.**
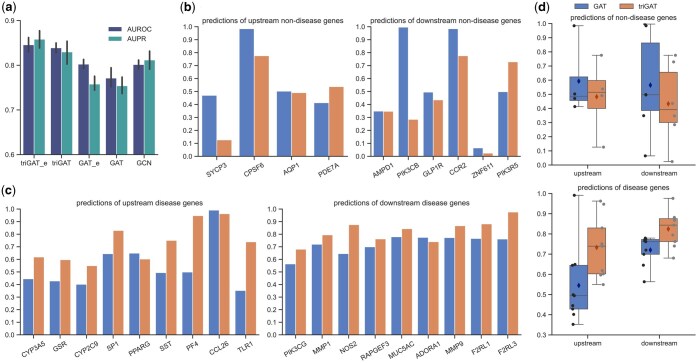
triGAT enables DGP-AMIO to precisely identify upstream and downstream disease genes. (a) Fivefold cross validation performance comparison of triGAT, GAT, and GCN. “_e” means edge features are included. (b)–(d), Predictions of triGAT and GAT on non-disease genes (b) and disease genes (c) in the test set which are located upstream and downstream of the disease genes in the training set, and the statistical distribution (d), where the diamond points represent mean values. The results of asthma are presented in this figure.

The digraph integrating directional gene regulatory relations contains more information than the undirected graph, which can be viewed as the weakened version of the digraph. We expect the model to integrate the direction information for accurate prediction. To prove that triGAT has better ability to extract structural features of digraph and predict disease genes, we performed experiments on asthma, using the disease genes collected from Malacards and expression data GSE143303. We compared triGAT with GAT and GCN based on the same graph. The input graph of triGAT and GAT was exactly the same while the input of GCN was the undirected graph derived from the original one. For fair comparison, the number of layers and node embedding dimensions are kept same for triGAT, GAT, and GCN. The AUROC and AUPR on the test set shows that triGAT is the best GNN model for predicting disease genes, followed by GCN and GAT (Fig. 3a). Although GAT receives the digraph as input, it yields the worst performance. We examined the disease-related probability of the disease genes in the test set predicted by GAT and found that the genes located downstream of known disease genes had higher probabilities than upstream disease genes (Fig. 3c and d). This phenomenon can be explained by the message passing mechanism in GAT, where message can only flow from upstream node to downstream node. GCN does not have this problem, which results in better performance than GAT, but also leads to a lack of directional information. triGAT enables messages to be passed from downstream to upstream while retaining the original digraph structure. Figure 3b–d shows that the upstream gene prediction accuracy of triGAT is significantly better than GAT and the downstream genes can also be more accurately predicted.

#### 3.1.3 DGP-AMIO benefits from the integration of multiple gene interaction databases and multi-omics

**Figure 4.**
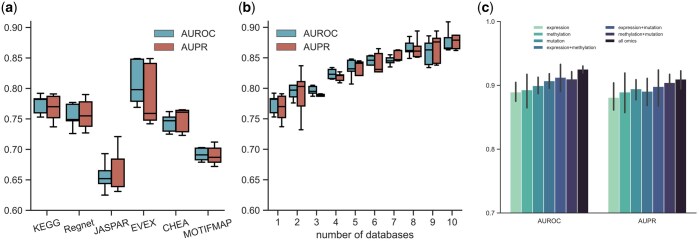
DGP-AMIO benefits from the integration of gene interaction databases and multi-omics. (a) Fivefold cross validation performances of DGP-AMIO trained on different single gene interaction network, where expression data and disease genes of breast cancer are used. (b) Fivefold cross validation performances of DGP-AMIO integrating different number of gene interaction networks from 1 to 10, where expression data and disease genes of breast cancer are used. (c) Fivefold cross validation performances of DGP-AMIO on colorectal cancer, using one type of omics, two types, and all three types (expression, DNA methylation, and gene mutation).

To evaluate whether DGP-AMIO trained on the graph integrating multiple databases was better than DGP-AMIO trained on the graph of a single database. We conducted experiments for breast cancer, given its high number of known disease genes available for training. We first trained DGP-AMIO on six single gene interaction networks. The AUROC and AUPR of DGP-AMIO fluctuated significantly with different single database (Fig. 4a), which suggests that for graph-based method, the accuracy of predicting disease genes is considerably affected by the gene interaction network used. Next, we trained DGP-AMIO on the integrated network. The results showed that as the number of integrated gene interaction databases increased (from 1 to 10, with the integration order: KEGG, RegNetwork, TRRUST, JASPAR, MOTIFMAP, EVEX, CHEA, STRING, CPDB, IREF), the AUROC, and AUPR of DGP-AMIO increased and is significantly higher than DGP-AMIO trained on any single database (Fig. 4b), which proved the effectiveness of multi-database integration. And to test whether the different integration orders significantly influence the performance trend as the number of databases increases, we test two more integration orders (Supplementary Fig. S4), and the increasing trend of performance does not change significantly. Furthermore, we trained DGP-AMIOs with and without database vectors on edges and compared their performances. Figure 3a shows that adding vectors containing database information on edges improved AUROC and AUPR of DGP-AMIO. This demonstrates that incorporating edge features into the training of attention coefficients enables the DGP-AMIO to learn the importance of different gene interaction data and allocate attention weights based on the database record. Overall, network integration provides more knowledge information for accurate disease gene prediction, and considering the limited number of known disease genes, using the integrated network maximizes the number of labeled samples for model training (Supplementary Fig. S6). These advantages of network integration enable DGP-AMIO to accurately identify disease genes.

Moreover, DGP-AMIO is a general framework that can integrate multi-omics data by simply concatenating multiple omics as node features. To explore whether multi-omics integration can improve the performance of DGP-AMIO, we downloaded from TCGA the expression, DNA methylation and gene mutation data, and trained DGP-AMIO using single type of omics, two types of omics and all three omics and evaluate their performances. Figure 4c is the results of colorectal cancer, showing that the AUROC and AUPR of DGP-AMIO using three omics is the highest, and using two omics is better than single omics. This demonstrates the multi-omics integration can further improve DGP-AMIO’s ability to predict disease genes. More multi-omics experiment results are in Supplementary Fig. S5.

### 3.2 Validation of newly predicted disease genes

#### 3.2.1 Validation based on external disease gene datasets

**Figure 5.**
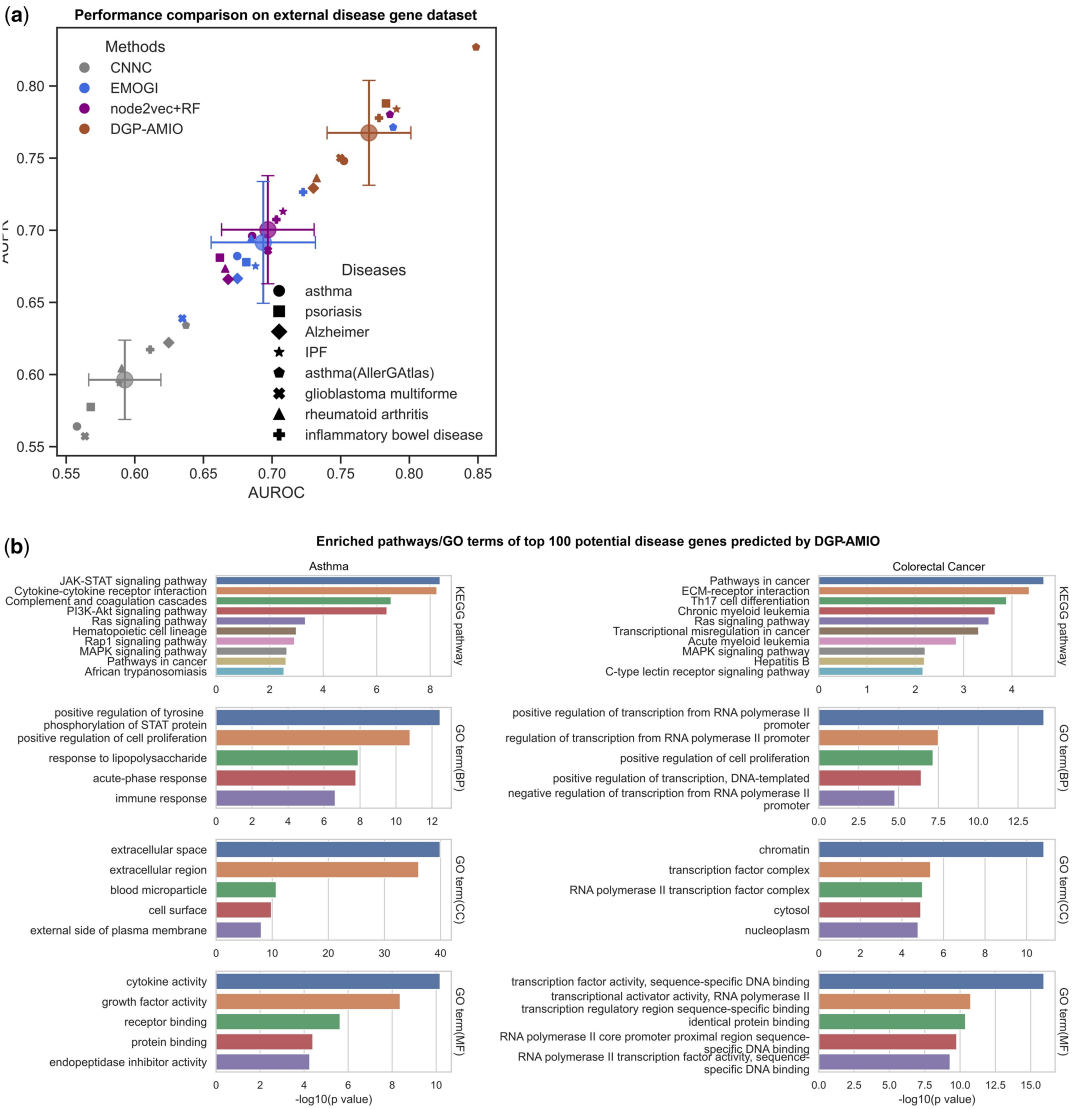
Validation of novel disease genes predicted by DGP-AMIO. (a) Performance comparison of DGP-AMIO and other methods on genes not included in the training and test set, collected from external disease gene datasets, DisGeNet, and AllerGAtlas. The horizontal and vertical axes represent AUROC and AUPR, respectively. The large circles correspond to average AUROCs and AUPRs across diseases for each method, and the crosses represent variances. (b) Enrichment analysis of top 100 genes predicted by DGP-AMIO (left: asthma, right: colorectal cancer), showing top 5 enriched GO terms of biological process (BP), cellular component (CC), molecular function (MF), and top 10 enriched KEGG pathways and their *P* values.

The disease genes used in the training, validation, and test set are collected from Malacards, while the labels of genes in the predictions of DGP-AMIO are unknown. To prove the reliability of DGP-AMIO’s predictions, we collected disease genes from DisGeNet (Piñero *et al.* 2017), a database of gene–disease associations from expert curated repositories, GWAS catalogues, animal models, and the literature, and for asthma we also collected disease genes from AllerGAtlas (Liu *et al.* 2018), a manually curated human allergy-related gene database. Then, we removed genes that appear in the training, validation, and test set. The remaining genes were labeled as “novel disease genes” and an equal number of genes were randomly sampled from the other unlabeled genes as negative samples. We calculated AUROC and AUPR based on these labeled genes and predictions and compared the performances between DGP-AMIO and other methods. The results in Fig. 5a show that DGP-AMIO achieves the highest AUROC and AUPR on external test sets of various diseases (closer to the top right corner of Fig. 5a compared to other methods). Results show that DGP-AMIO outperforms other methods in predicting novel disease genes not appearing in the training and test set, demonstrating the superior potential of DGP-AMIO in discovering novel disease genes.

#### 3.2.2 Enrichment analysis

To further validate that the top genes predicted by DGP-AMIO are disease-related, we performed gene enrichment analysis on the top 100 genes of DGP-AMIO's predictions. Figure 5b shows the enrichment result of asthma and colorectal cancer, listing top 5 significantly enriched GO terms of biological process (BP), cellular component (CC), molecular function (MF) and top 10 significantly enriched KEGG pathways and the corresponding *P* values (transformed by -log10).

For asthma, the most significantly enriched GO terms are “positive regulation of tyrosine phosphorylation of STAT protein,” “positive regulation of cell proliferation,” “extracellular space,” “extracellular region,” “cytokine activity,” and “growth factor activity,” all of which are related to asthma. Protein tyrosine phosphorylation of STAT is involved in regulating the immune response and its inhibition can reduce the development of asthma (Pouliot *et al.* 2009). The cell proliferation of airway smooth muscle cells stimulated by airway inflammation is recognized to be crucial to asthma (Johnson *et al.* 2001, Khan 2013). “Extracellular space” and “extracellular region” are asthma-related because extracellular vesicles, matrix components and DNA play an important role in the pathogenesis of asthma (Alashkar Alhamwe *et al.* 2021). Cytokine activity is closely associated with asthma, as cytokines like IL-4, IL-5, and IL-13 can promote mucus overproduction and bronchial hyperresponsiveness (Lambrecht *et al.* 2019), which are symptoms of asthma. Many growth factors are also involved in asthma such as EGF, FGF, and TGFs (Kardas *et al.* 2020). Among the enriched KEGG pathways, “JAK-STAT signaling pathway,” “cytokine-cytokine receptor interaction,” “complement and coagulation cascades,” and “PI3K-Akt signaling pathway” have the lowest *P* value. JAK-STAT signaling pathway transduces cytokine-mediated signals which are pivotal in the development of asthma (Pernis and Rothman 2002), and targeting this pathway has been verified to have a therapeutic effect on asthma (Vale 2016). Cytokine-cytokine receptor interaction is enriched because of the vital role of cytokines in asthma discussed above. Complement and coagulation system is found to be in association with asthma control (Kokelj *et al.* 2023). The PI3K-Akt signaling pathway can promote airway inflammation and hyperresponsiveness (Medina‐Tato *et al.* 2007), therefore is crucial in asthma.

For colorectal cancer, the top enriched GO terms are “regulation of transcription from RNA polymerase II,” “positive regulation of cell proliferation,” “chromatin,” “transcription factor complex,” “transcription factor activity,” and “transcription activator activity,” which are all cancer-related. RNA polymerase II is related to disruption of transcription elongation which is implicated in cancer (Cermakova *et al.* 2021). The abnormal cell proliferation is the main cause of various cancer. “Chromatin” is cancer-related as its dysregulation is linked to mutation and cancer (Sehgal and Chaturvedi 2023). Cancer development requires constitutive expression/activation of transcription factors (TFs) and activators for growth and survival (Vishnoi *et al.* 2020) and many TFs are tumor suppressors or oncogenes. The most significantly enriched KEGG pathways are “pathways in cancer” (evidently cancer-related), “ECM-receptor interaction” and “Th17 cell differentiation.” The ECM receptors constitute crucial pathways involved in colorectal cancer progression and metastasis (Nersisyan *et al.* 2021) and Th17 cells are also highly related to colorectal cancer progression.

In conclusion, the top genes predicted by DGP-AMIO are significantly enriched in disease-related GO terms and pathways, demonstrating DGP-AMIO’s ability of discovering novel disease genes.

#### 3.2.3 Relevant research about the predicted unknown genes

We listed the top 10 potential disease genes of asthma and colorectal cancer predicted by DGP-AMIO, as shown in Table 1. We searched them online and found that most of them were studied as disease-related genes in the previous research, which indicates the prediction of DGP-AMIO is consistent with the existing studies. This demonstrates that DGP-AMIO is an effective computational method for discovering novel disease genes.

**Table 1.** Top 10 ranked genes of asthma and colorectal cancer predicted by DGP-AMIO and their functions in disease supported by relevant studies.

Gene	Function	Refs.
*Asthma*		
HBB	Potential biomarker for asthma diagnosis	Zhou *et al.* (2023)
CEACAM7	CEACAM7 gene expression was increased in severe asthma	Shikotra *et al.* (2017)
LIF	LIF seems to be required for airway inflammation and central sensitization in asthma	Lin *et al.* (2014)
EPO	EPO plays an important role in the pathogenesis of asthma by mediating oxidative events	Wu *et al.* (2022)
LYZ	An immune-related gene which has an important antibacterial activity in the airway epithelium and is expressed in allergic asthma	Pan *et al.* (2023) and Sun *et al.* (2020)
OSM	OSM expression drives airway inflammatory and mucus secretion in severe asthma	Headland *et al.* (2022)
REN	REN encodes renin, a part of the renin-angiotensin system which is activated in asthma	Chiba *et al.* (2023)
SFTPA1	SFTPA1 is involved in inflammation mechanism and its variation is associated with acute and chronic lung diseases including asthma	Bouma *et al.* (2023) and Silveyra and Floros (2013)
CD34	CD34 enhances mast-cell and eosinophil invasiveness and its expression by these cells is a prerequisite for development of allergic asthma	Blanchet *et al.* (2007)
PLAT	potential severe asthma-related gene	Cheng *et al.* (2023)
*Colorectal cancer*		
RMRP	Oncogene which inactivates p53 in colorectal cancer	Chen *et al.* (2021)
MYLK-AS1	Prognostic marker for colon adenocarcinoma	Xing *et al.* (2018)
FGD5-AS1	FGD5-AS1 promotes colorectal cancer cell proliferation, migration, and invasion	Li *et al.* (2019)
E2F5	Silencing E2F5 can inhibit the growth of SW-948, a colon cancer cell line	Yang *et al.* (2019)
HSPA1A	Upregulation of HSPA1A/HSPA1B is related to poor survival in colon cancer	Guan *et al.* (2021)
HSPA1B		
GATA6	GATA6 promotes colon cancer cell invasion and enhances colon cancer cells’ stemness	Belaguli *et al.* (2010) and Lai *et al.* (2020)
HOXA4	Overexpression of HOXA4 contributes to colon cancer cell overpopulation	Bhatlekar *et al.* (2018)
COL1A2	A tumor suppressor in CRC, regulating numerous oncogenes	Yu *et al.* (2018)
FGFBP1	Upregulation of FGFBP1 promotes CRC cell migration and invasion ability	Chen *et al.* (2020)

## 4 Discussion

In this work, we proposed DGP-AMIO, a GAT-based disease gene predictor with integration of gene interaction networks from different databases and multi-omics. We demonstrated DGP-AMIO has a higher accuracy and generalization than existing methods across 20 disease datasets, with AUROC and AUPR of most diseases reaching above 0.9 on test set. Furthermore, we conducted enrichment analysis and literature search on the top genes predicted by DGP-AMIO, with results showing most enriched GO terms/pathways and top genes are disease-related supported by existing literature. This provides compelling evidence for the efficacy and accuracy of our method as a tool for predicting novel disease-associated genes.

DGP-AMIO enhanced its predictive performance by improving the processing of directed gene interaction relationships. This demonstrates the significance of directional information in disease and biological contexts, particularly in the regulatory directionality within gene interaction networks. Furthermore, the integration of more comprehensive gene interaction data and multi-omics data by DGP-AMIO has facilitated precise predictions, indicating the diversity and complexity of disease mechanisms. This highlights the existence of numerous unknown disease mechanisms that necessitate exploration and discovery. Lastly, the model's predictions have been extensively validated on existing biological research and functional data, affirming the potential of computational tools in accelerating the discovery of disease genes and enhancing our understanding of disease mechanisms.

We acknowledge several limitations of DGP-AMIO. We used random sampling from nonknown disease genes as negative samples for training. Experimental observations revealed that different sampling can influence DGP-AMIO’s performance, highlighting the need for improved negative sample construction strategies. The quality of omics data used as input can also affect the performance. Additionally, DGP-AMIO exhibits limited predictive performance for diseases with a scarce number of known disease genes documented in Malacards. Regarding the aforementioned issues, future improvement work will focus on the following aspects: (i) Developing more appropriate strategies for constructing negative samples, including but not limited to, introducing constraints during sampling and incorporating information from relevant studies. (i) Utilizing gene embeddings obtained from large models pretrained on large-scale transcriptomic data. (iii) Augmenting the scale of labeled samples by integrating literature data and clinical trial data. In summary, DGP-AMIO is a general computational approach capable of accurately predicting disease genes, holding significant importance and practical value in accelerating human understanding of disease mechanisms and drug development.

## Supplementary Material

btaf181_Supplementary_Data

## Data Availability

Gene expression data are available at GEO (https://www.ncbi.nlm.nih.gov/geo/) and the accession numbers are listed in Supplementary Table S4. All gene interaction networks are available at public databases, whose websites are listed in Supplementary Section S2.9. Human disease genes are available at Malacards (https://www.malacards.org/). The code and data to train DGP-AMIO, evaluate model performance and predict disease genes is available at https://github.com/yangkaiyuan1027/DGP-AMIO and https://zenodo.org/records/15081814 (DOI: 10.5281/zenodo.15081814).
