# Supplementary material for: Integration of multi-source gene interaction networks and omics data with graph attention networks to identify novel disease genes
Source: Bioinformatics. 2025 Apr 23;41(7):btaf181. doi: 10.1093/bioinformatics/btaf181 (PMC12270254; doi:10.1093/bioinformatics/btaf181)
Supplement: btaf181_Supplementary_Data [file btaf181_supplementary_data.zip › Supplementary Information.docx]

Supplementary Materials

## 1 Results

### 1.1 Hyper-Parameter Optimization


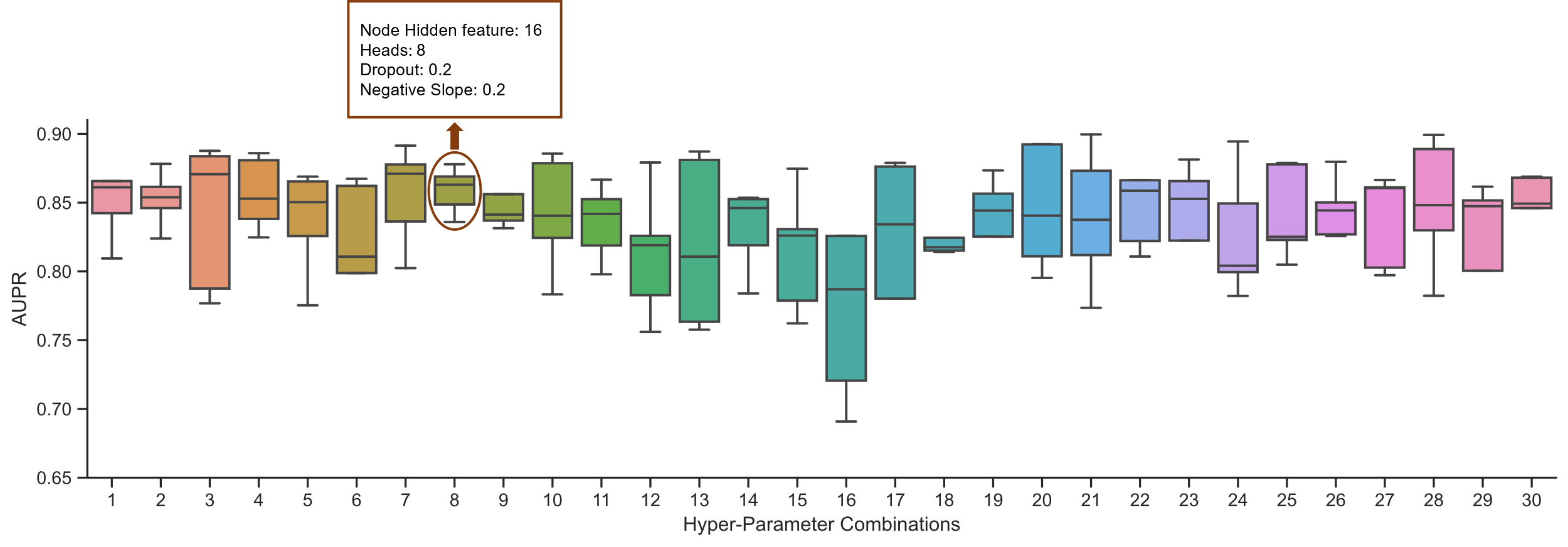


Figure S1: **The best performing model hyper-parameters combination.** We performed a grid search among 360 combinations of model hyperparameters using breast cancer data. To consider both performance and robustness, we conducted five-fold cross validation on the training set and calculated the mean and variance of the AUPR. The optimal hyperparameter combination we selected is: node hidden feature of 16, heads of 8, a dropout rate of 0.2 and a negative slope of 0.2 (circled in the figure). This set of model hyperparameters strikes the best balance between performance and robustness, with the second highest average AUPR and the third lowest variance.


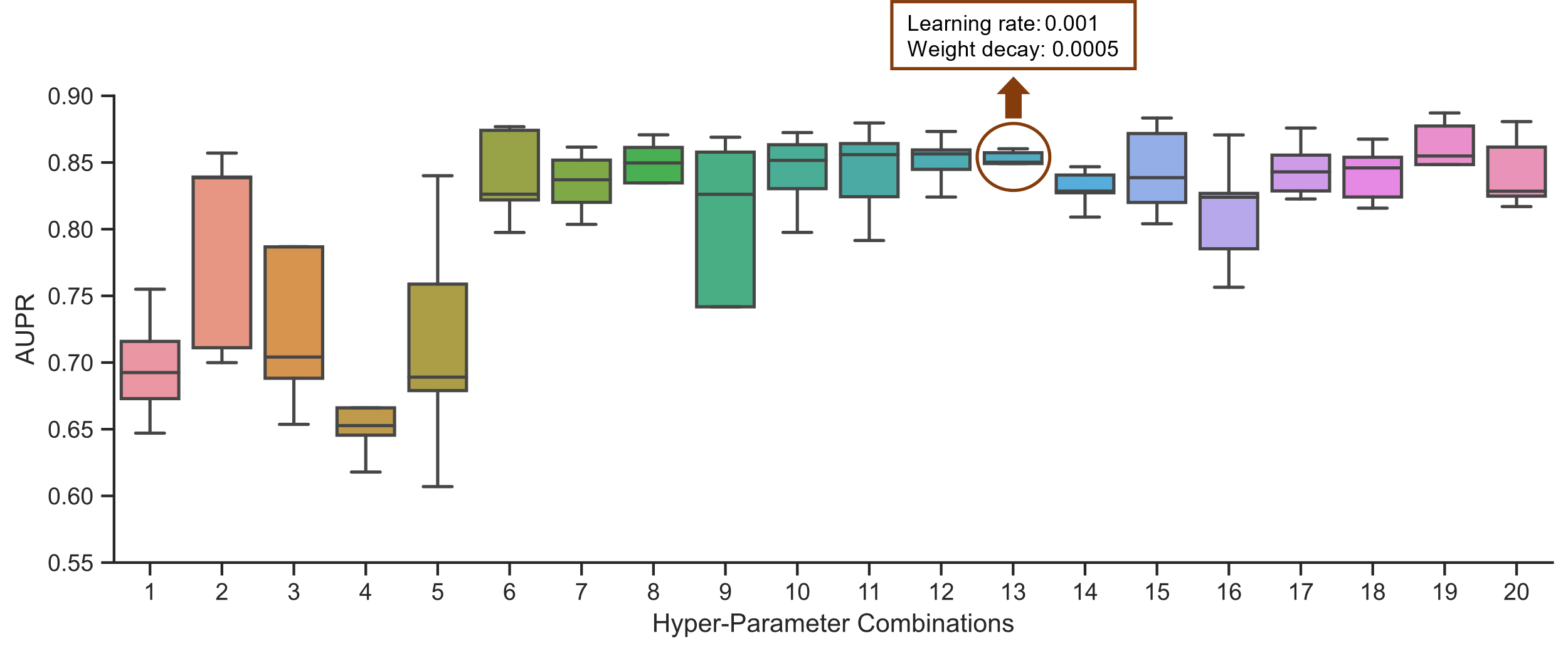


Figure S2: **The best performing optimizer hyper-parameters combination.** After determining the optimal model hyper-parameter combinations, we further performed a grid search to find the optimal hyper-parameters of ADAM optimizer we utilized. We fixed all model hyper-parameters with the optimal combinations according to Figure S1. Then similarly we conducted a five-fold cross validation on training set and evaluate the performance and robustness with the average AUPR and the variance. The optimal optimizer hyper-parameter we selected is: a learning rate of 0.001 and a weight decay of 0.0005 (circled in the figure), with the third highest average AUPR and the lowest variance.

### 1.2 Performance Comparison

**
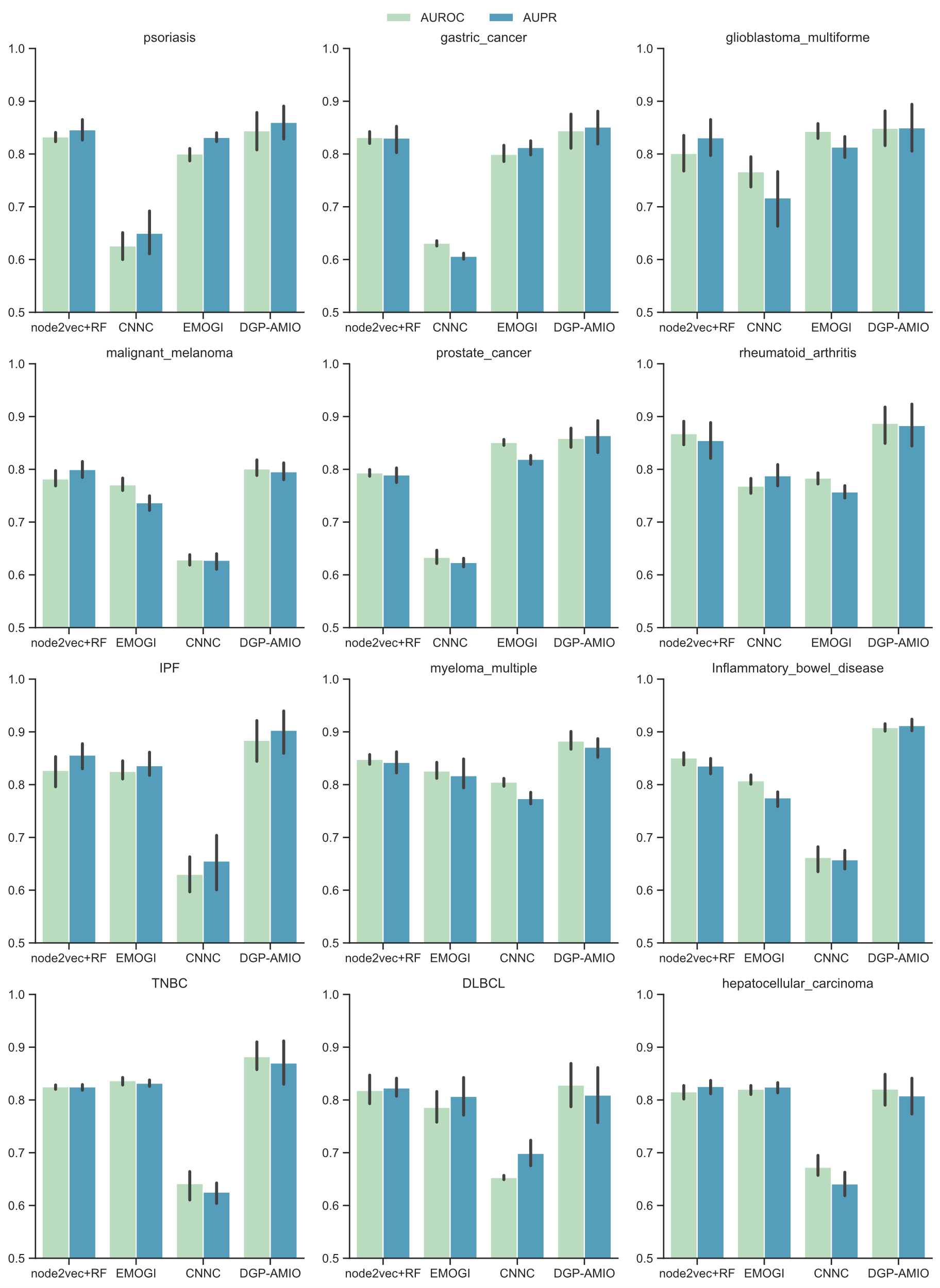
**

Figure S3: **Performance comparison on datasets of different diseases.** For each method we performed five-fold cross validation to train five ensembles based on the training set and calculated AUROCs and AUPRs on the test set (see subsection 2.4 Training and Performance Evaluation). And for each disease we sampled five different subsets of negative samples and performed cross validation process separately.

### 1.3 Different Orders of Gene Interaction Network Integration


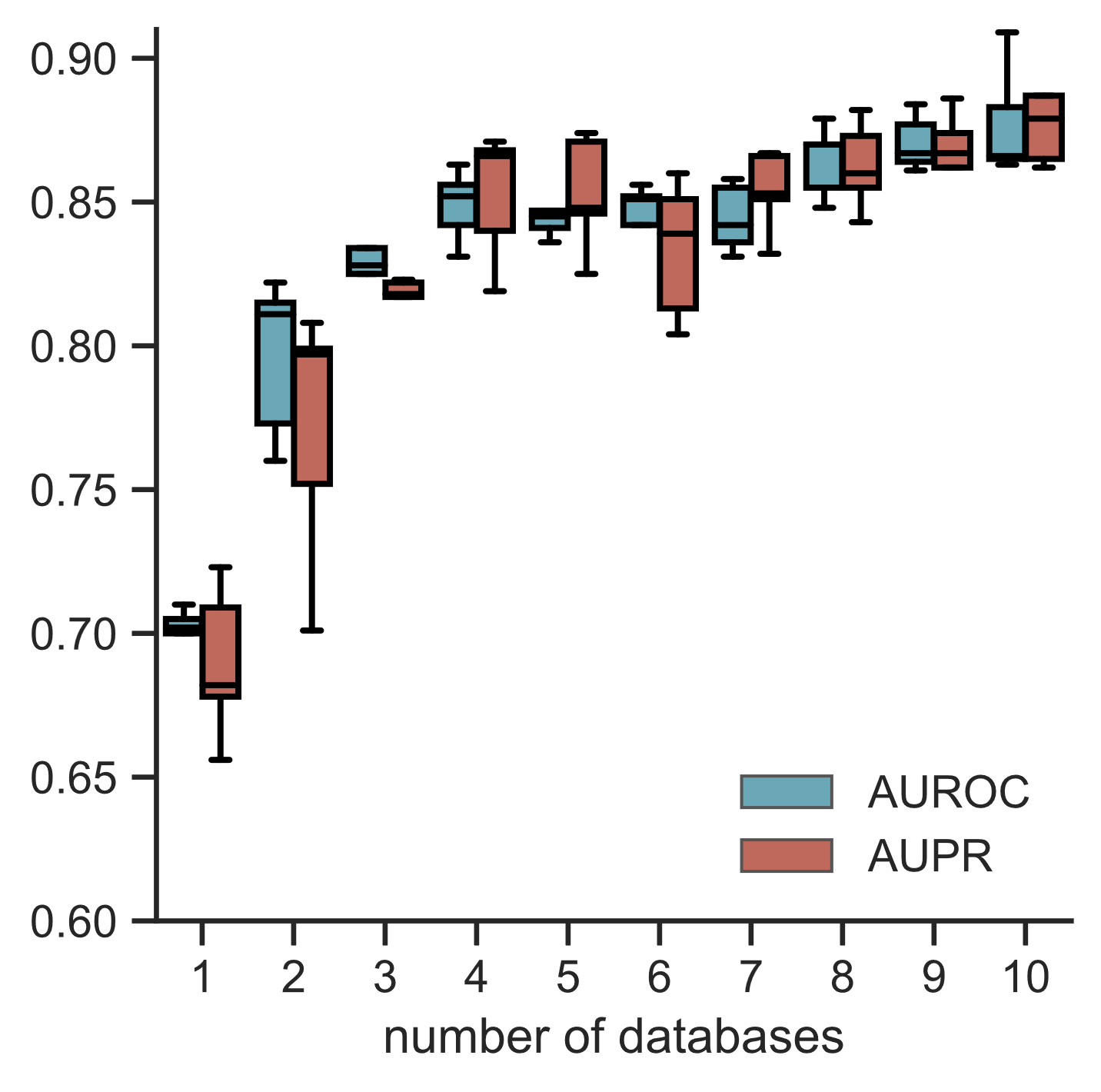

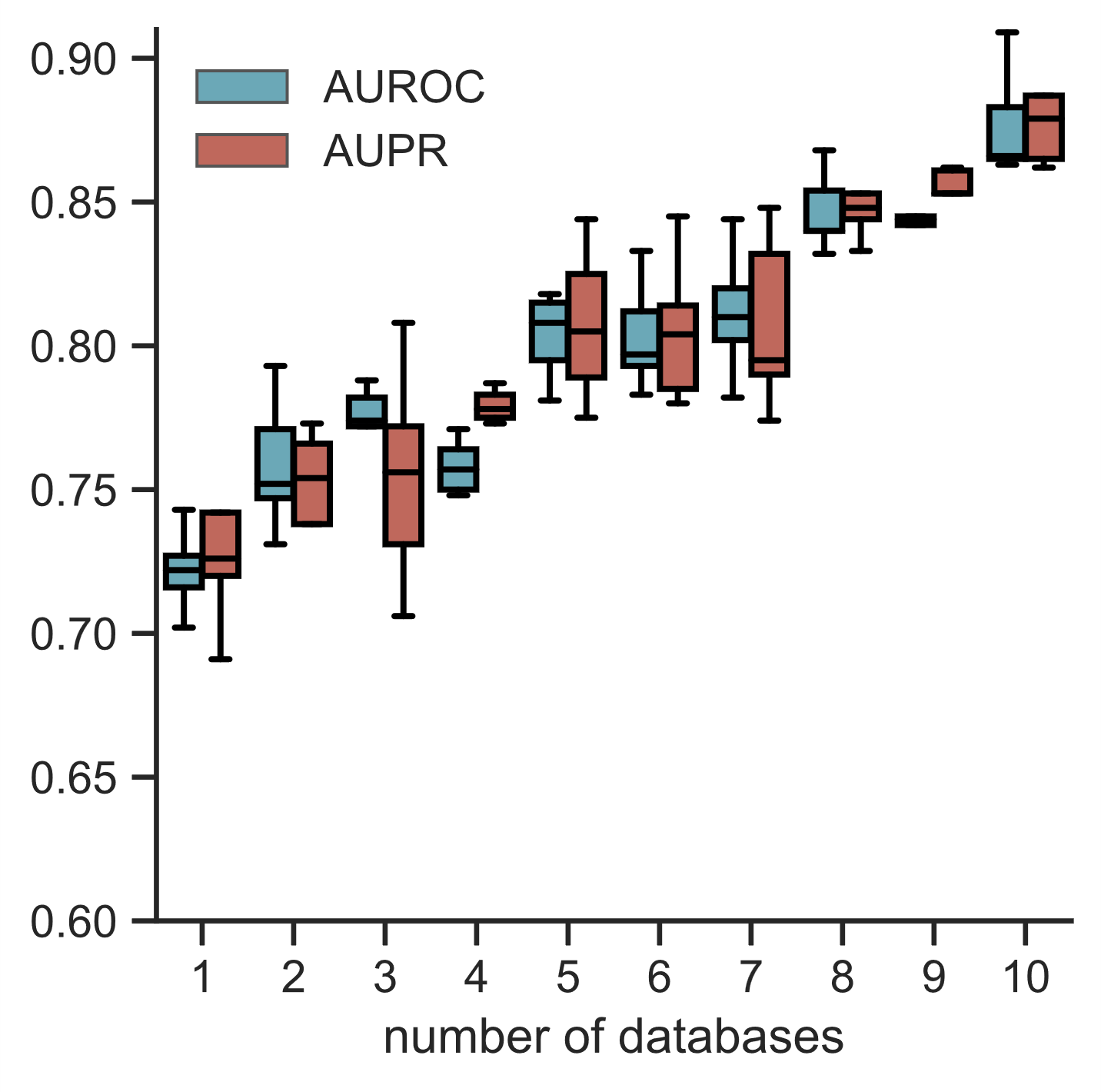


Figure S4: To test whether the different integration orders significantly influence the performance trend as the number of databases increases, we test two more integration orders. (Left: trrust, EVEX, RegNetwork, CHEA, KEGG, IREF, JASPAR, string, MOTIFMAP, CPDB. Right: IREF, CHEA, JASPAR, trrust, KEGG, MOTIFMAP, Regnetwork, string, CPDB, EVEX).

### 1.4 Multi-omics Integration Experiment


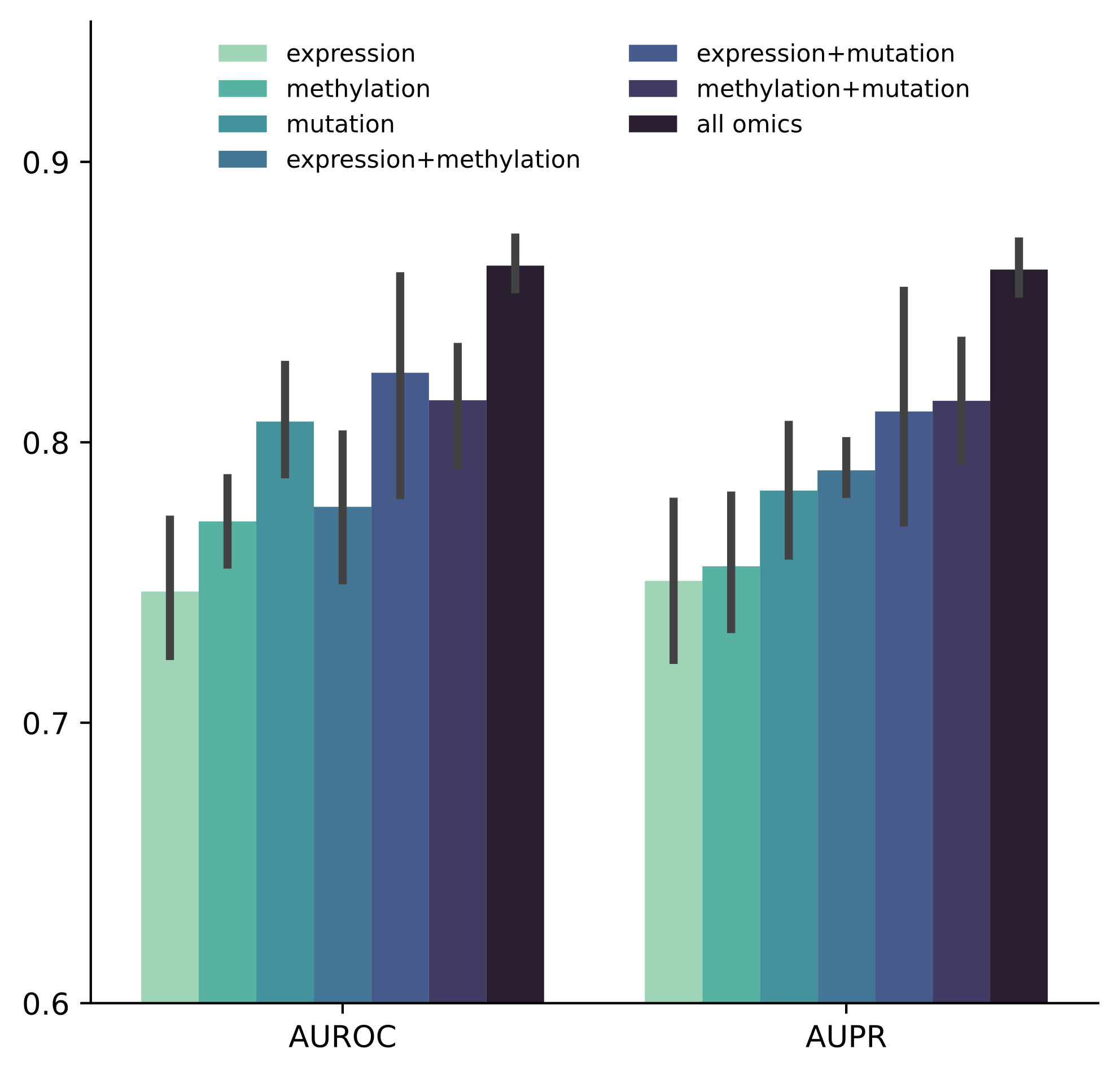

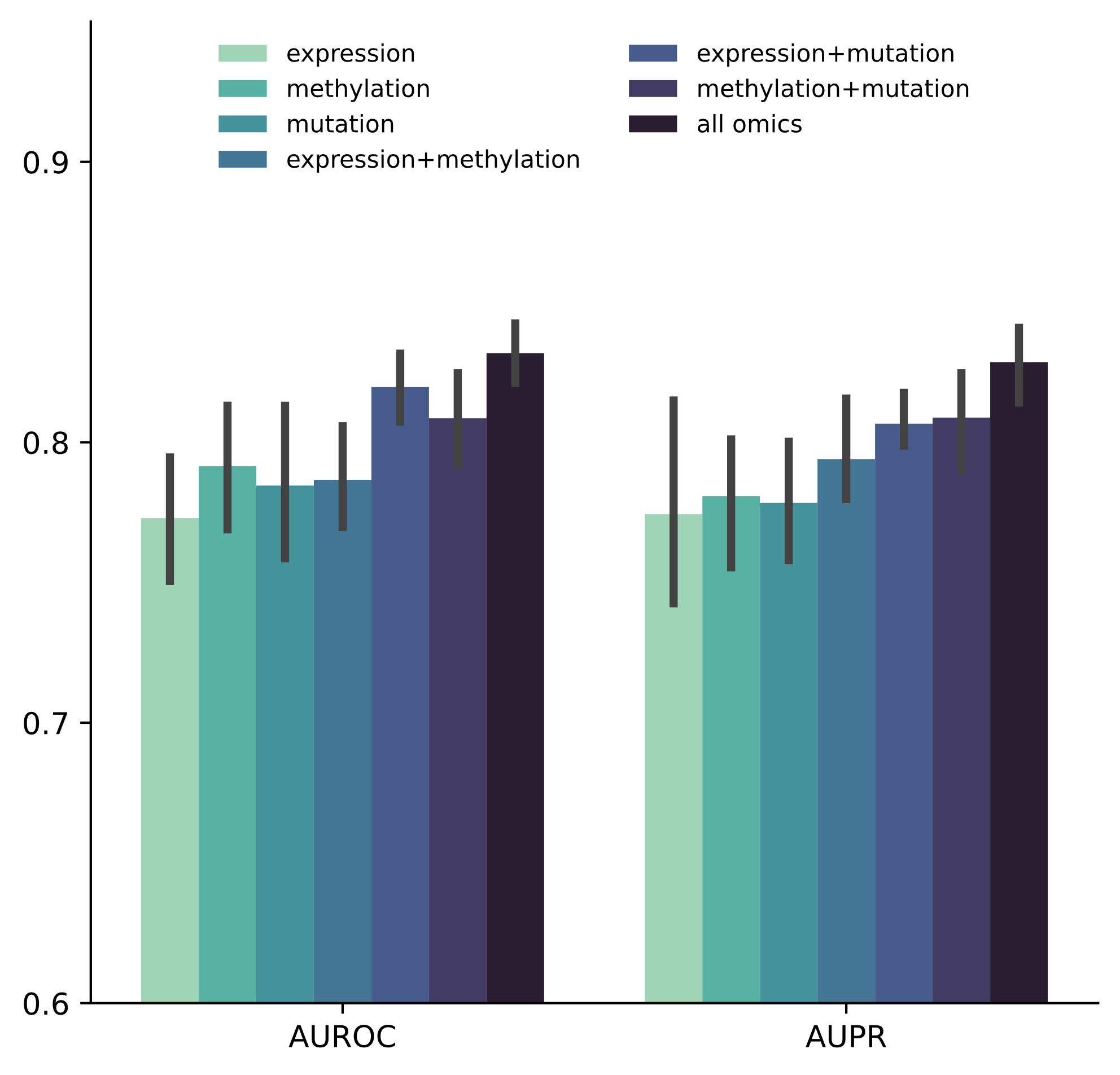


Figure S5: Muti-omics experiments (Left: breast cancer. Right: lung cancer)

### 1.5 Known Disease Genes in Different Networks


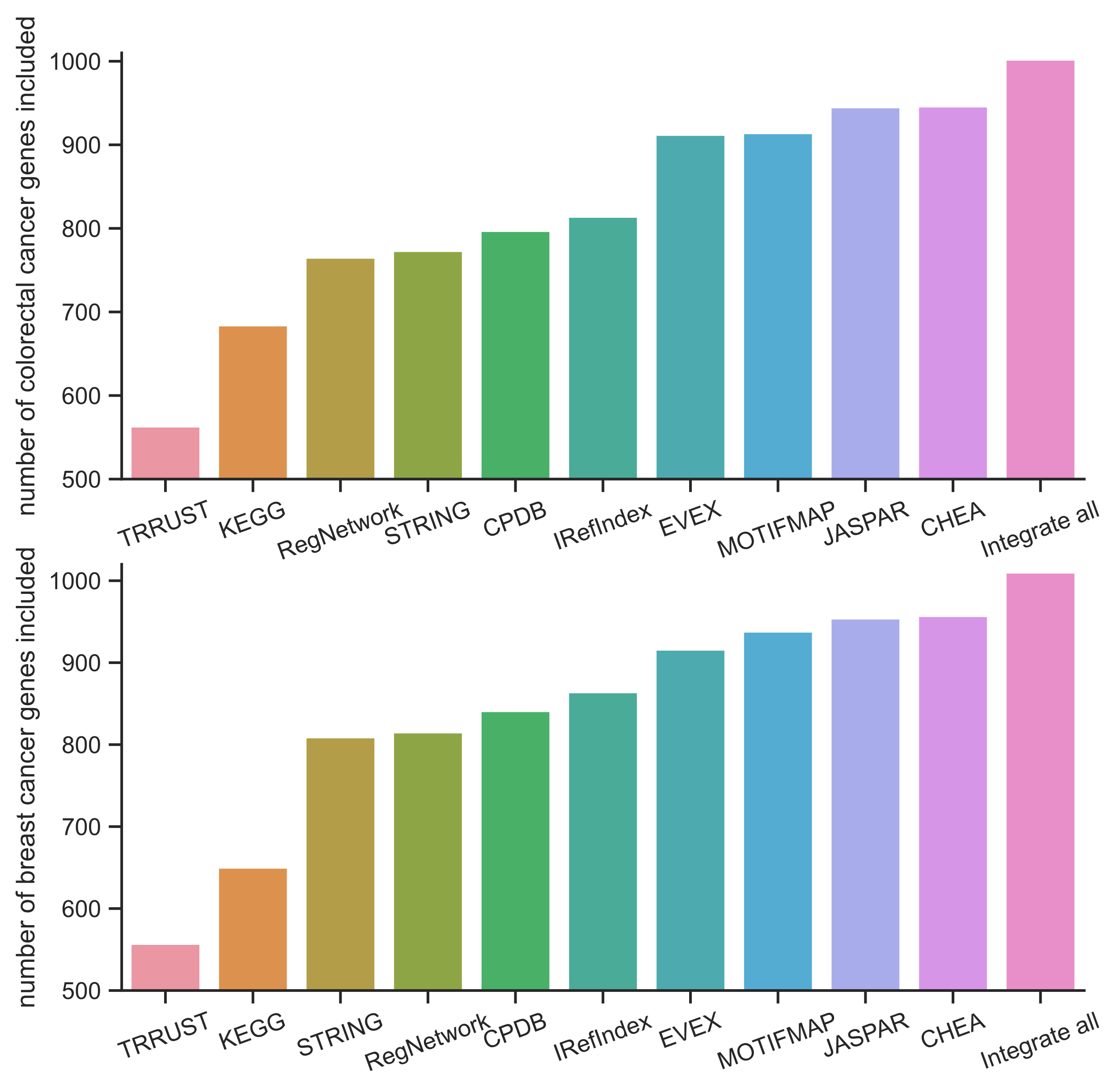


**b**

**a**

Figure S6: **Number of known disease genes included in different gene interaction networks** (a: colorectal cancer, b: breast cancer). Known disease genes represent only a small fraction of human genome, even for diseases with relatively larger numbers of associated genes such as breast cancer and colorectal cancer, which have approximately a thousand relevant genes. This limited availability of labeled data poses a challenge for GNN-based methods, as they require an overlap between the graph and the labeled samples. Constructing the graph based on a single gene interaction network results in the loss of a number of labeled samples, especially for small-scale gene interaction networks like TRRUST and KEGG. DGP-AMIO effectively addresses this issue by integrating multiple gene interaction networks from diverse databases. This integration enables DGP-AMIO to have the maximum number of labeled samples to train compared to using a single gene interaction network, resulting in more accurate prediction.

### 1.6 Visualization of DGP-AMIO’s predictions in the graph

**
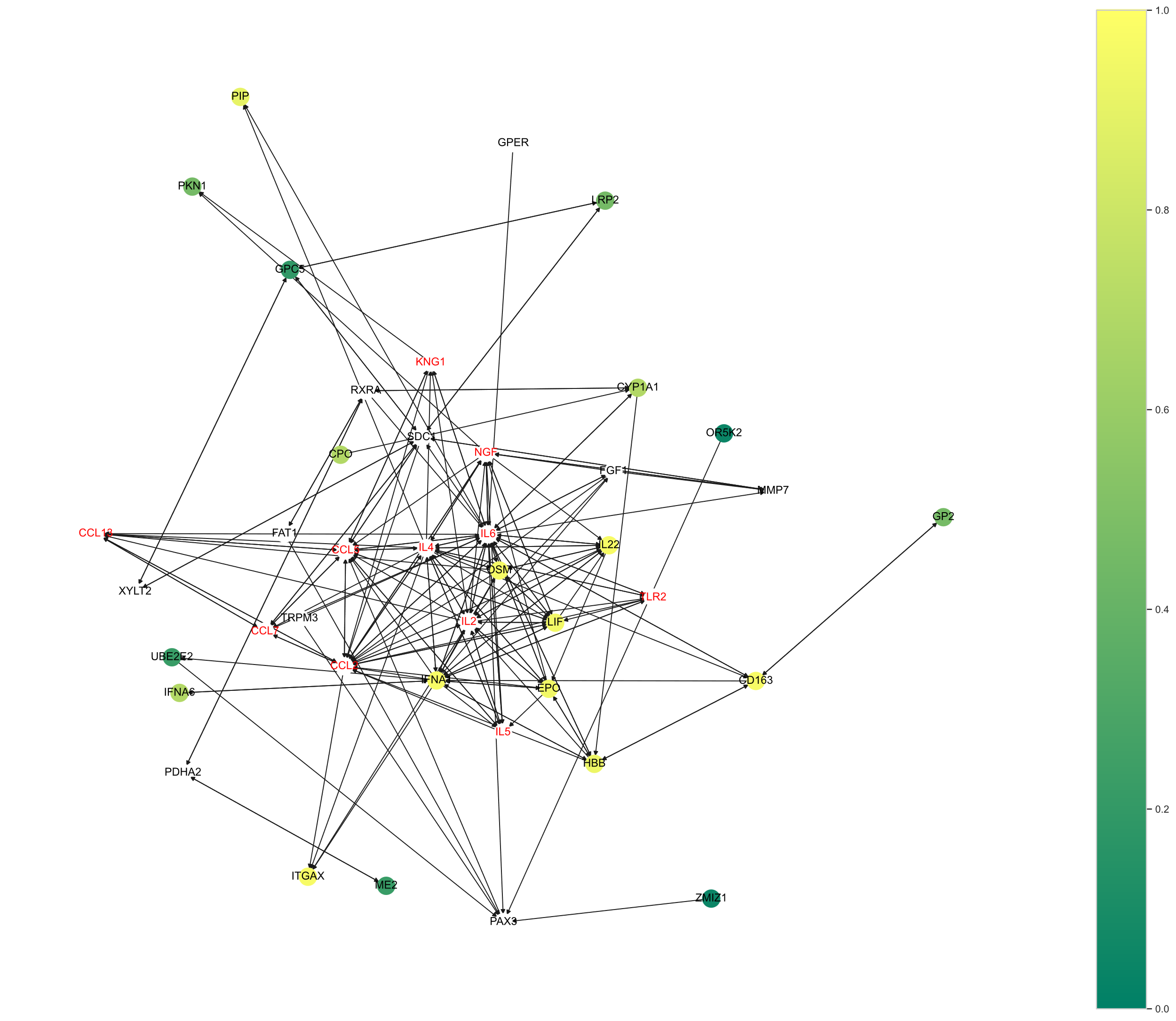
**

Figure S7: **Predictions of DGP-AMIO on the asthma dataset and the interactions between the gene to be predicted and known asthma genes.** We visualized the predictive results of certain genes and their interactions with a subset of known asthma genes in a graph. Asthma genes (like IL4, IL6, NGF) and non-asthma genes (like GPER, FAT1, MMP7) from the training set are represented by nodes of red text and black text on a white background, respectively. The probability of a gene being predicted as an asthma gene by DGP-AMIO is represented by a gradient color. It can be observed that genes with a high probability of being related with asthma exhibit close interactions with known asthma genes, like OSM, LIF, EPO, HBB, while genes with a low probability of being related with asthma display sparse interactions with known asthma genes.

### 1.7 Enrichment Analysis


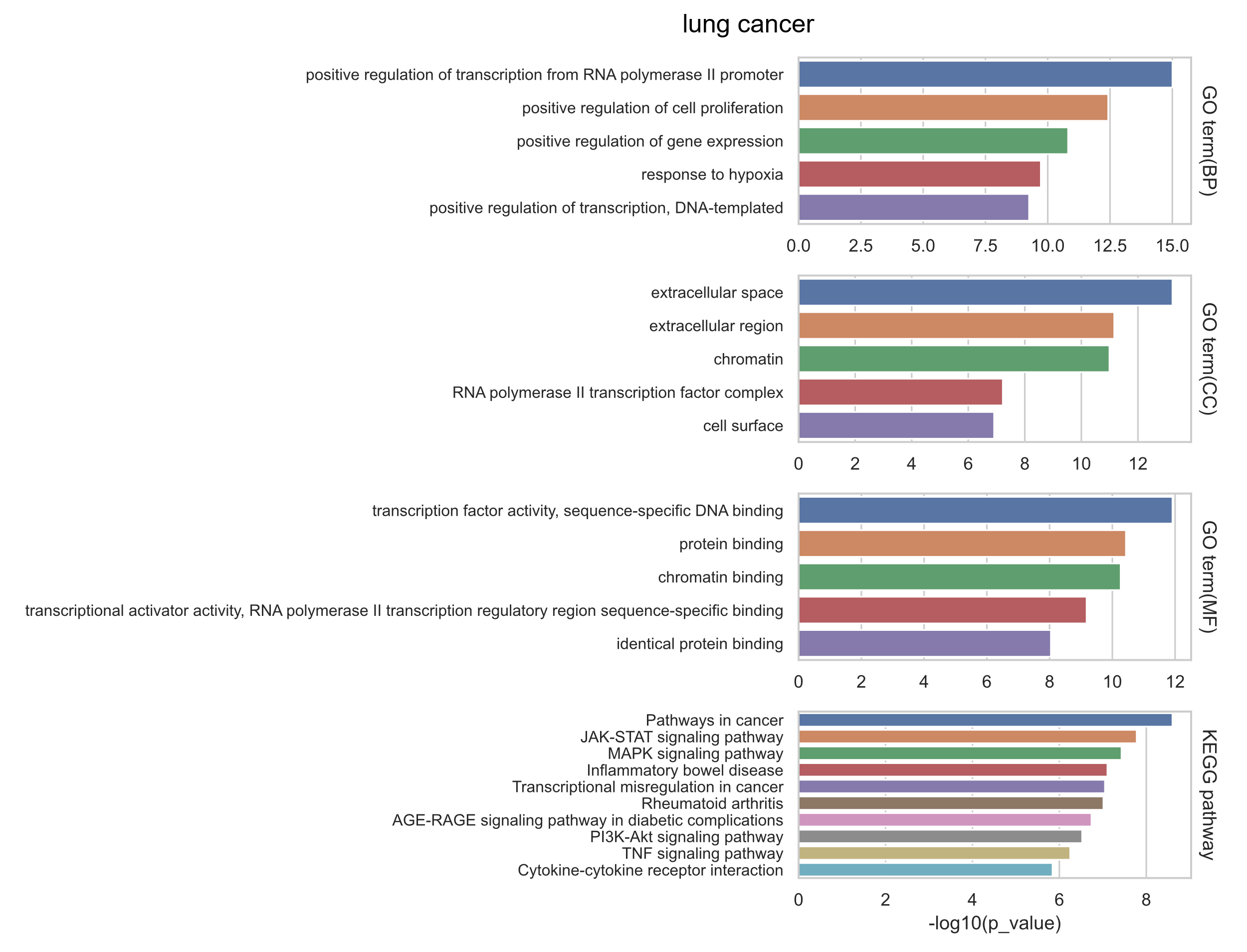


Figure S8: **Enrichment analysis of top 100 genes predicted by DGP-AMIO (results of lung cancer)**, listing top 5 significantly enriched GO terms of biological process (BP), cellular component (CC), molecular function (MF) and top 10 enriched KEGG pathways and the corresponding p values.


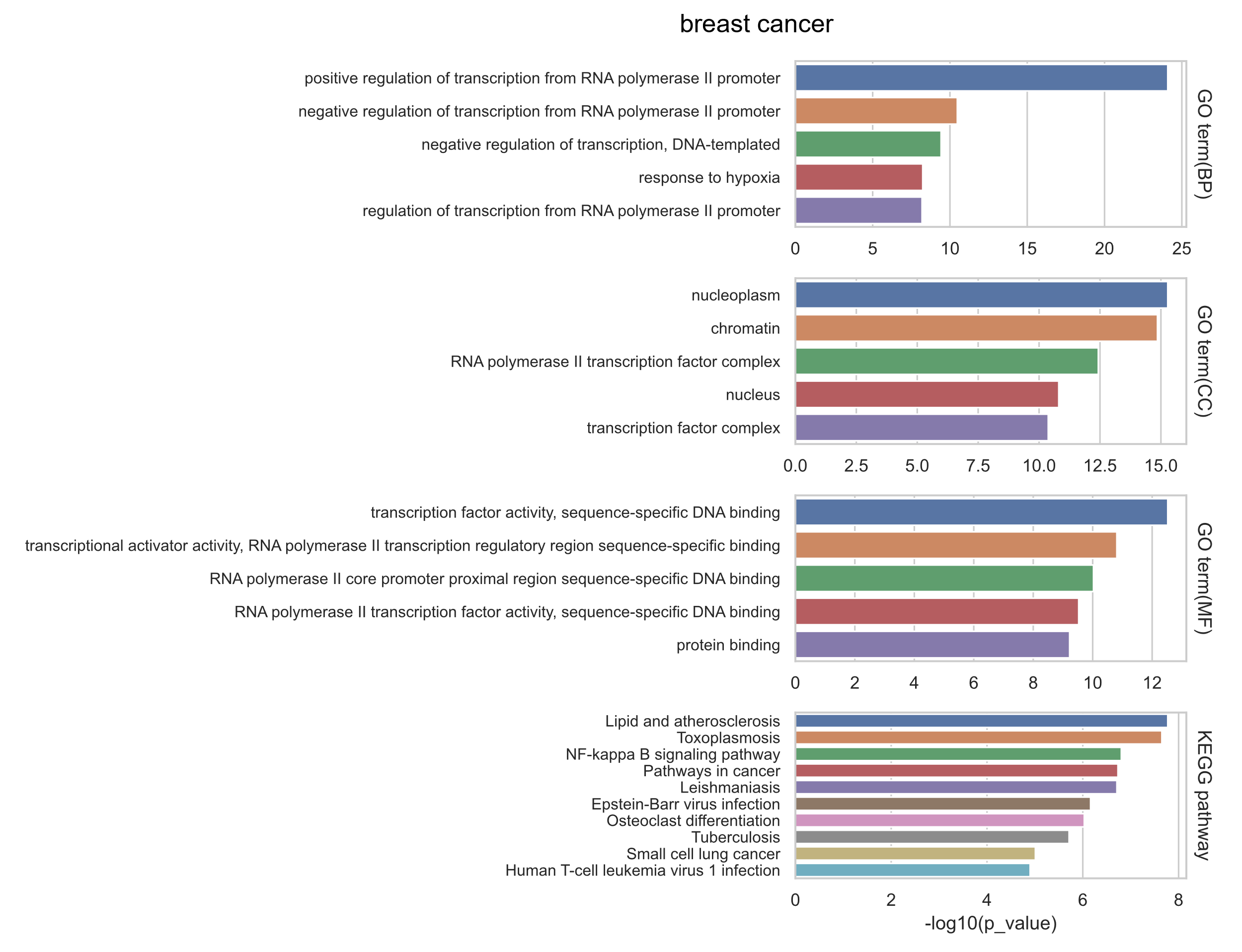


Figure S9: **Enrichment analysis of top 100 genes predicted by DGP-AMIO (results of breast cancer)**

### 1.8 Newly Predicted Disease Genes

Table S1: **Top 30 genes ranked by the probability of being disease genes predicted by DGP-AMIO.** (results of asthma, Alzheimer, inflammatory bowel disease, rheumatoid arthritis and breast cancer)

|  | Asthma | Alzheimer | Inflammatory bowel disease | Rheumatoid arthritis | Breast cancer |
| --- | --- | --- | --- | --- | --- |
| 1 | HBB | IFNG | CEACAM7 | IL8 | E2F2 |
| 2 | CEACAM7 | TGFB1 | COL1A1 | TLR6 | TFDP1 |
| 3 | LIF | FGF2 | MMP12 | CCL1 | HSF1 |
| 4 | EPO | EGF | MUC8 | CD163 | CD14 |
| 5 | LYZ | VEGFA | COL1A2 | IL1RL2 | TBP |
| 6 | OSM | POMC | MT1F | NFKBIZ | EGR1 |
| 7 | REN | BMP2 | ANPEP | FIGF | ARNT |
| 8 | SFTPA1 | MMP2 | REG1A | IRF6 | IFNG |
| 9 | CD34 | P4HA2 | TSLP | CSF3 | YY1 |
| 10 | PLAT | MBP | HBB | CCL7 | ATF3 |
| 11 | PIP | MMP9 | IL33 | DARC | CD40 |
| 12 | TH1L | TNFSF10 | PRSS55 | SELP | EPHB2 |
| 13 | PLG | PLAT | TNFRSF11A | CAMP | STAT4 |
| 14 | DARC | CD40LG | COL3A1 | IL12B | KAT2B |
| 15 | CD38 | HIF1A | CSF2 | LBP | SOCS3 |
| 16 | HGF | MMP1 | NFKBIZ | TNFSF12 | WNK2 |
| 17 | IGSF1 | CDH1 | OSM | CCL8 | HNF4A |
| 18 | TFPI | HSP90AB1 | IL32 | CCL15 | GATA1 |
| 19 | HBA1 | ERBB2 | CSF3 | CD58 | PLAT |
| 20 | ITGAX | CDKN1A | DEFA4 | ITGAX | TLR4 |
| 21 | CRP | BGN | SLC35G3 | TPO | BCR |
| 22 | CD8A | IL10 | JUNB | IL12A | PPARA |
| 23 | IL10RA | MOK | PLAU | CCL13 | MYCN |
| 24 | C4BPA | HGF | TIMP2 | FCGR1C | HDAC3 |
| 25 | PRL | VIM | CCL26 | KIAA0101 | IRF8 |
| 26 | SERPINF2 | WDR83 | COL6A5 | TLR8 | IL6ST |
| 27 | THBD | MET | ITGAX | F3 | CEBPB |
| 28 | CCL1 | CD44 | HP | MMP27 | IL15 |
| 29 | IL8 | UBE3C | CCL17 | IL12RB1 | IL1RN |
| 30 | CXCR7 | IL4 | CD163 | WNK2 | BTRC |

### 1.9 Robustness Experiments

Table S2: To test the robustness of DGP-AMIO when the label of disease genes in the training set is not accurate, we randomly sampled some false positive (FP) samples and added them in the training set. From the results we can see that the model exhibits good robustness, as even with an increase in false positives to 100, the AUROC and AUPR only decrease by less than 0.02

| Number of FP | 0 | 20 | 40 | 60 | 80 | 100 | 200 |
| --- | --- | --- | --- | --- | --- | --- | --- |
| AUROC | 0.873 | 0.871 | 0.866 | 0.871 | 0.861 | 0.860 | 0.850 |
| AUPR | 0.884 | 0.880 | 0.877 | 0.877 | 0.869 | 0.865 | 0.859 |
| Number of FP | 400 | 800 |  |  |  |  |  |
| AUROC | 0.833 | 0.818 |  |  |  |  |  |
| AUPR | 0.849 | 0.815 |  |  |  |  |  |

### 1.10 Node2vec Dimension Optimization

Table S3: To find the best performing dimension numbers of node2vec, we test different dimensions of node embeddings learned by node2vec on breast cancer.

| Dimension | 4 | 8 | 16 | **32** | 64 |
| --- | --- | --- | --- | --- | --- |
| AUROC | 0.802 | 0.805 | 0.832 | **0.844** | 0.838 |
| AUPR | 0.800 | 0.821 | 0.851 | **0.860** | 0.840 |

## 2 Methods & Materials

### 2.1 Metrics

To evaluate the performance of DGP-AMIO and other methods, we computed two commonly used metrics for evaluating the performance of binary classifiers: AUROC (Area Under the Receiver Operating Characteristic curve) and AUPR (Area Under the Precision-Recall curve). Based on the continuous probability values given by the classifier, pairs of True Positive Rate (TPR)-False Positive Rate (FPR) and Precision-Recall can be computed for all possible thresholds. Then TPR-FPR and P-R curves can be plotted, and the area under the curves represents the AUROC and AUPR values. Both AUROC and AUPR range from 0 to 1, with values closer to 1 indicating better model performance.

Considering that AUROC can be influenced by imbalanced class distributions of the dataset, we randomly sampled an equal number of genes from the unknown genes as negative samples, ensuring the class balance.

### 2.2 Graph Integration

In this section, we provide a schematic diagram that explain in detail how DGP-AMIO integrates multiple gene interaction networks and generates the edge feature matrix representing database information, shown as Figure S10. For example, in Figure S10, we integrated three gene networks, so the dimension of edge feature is three, and the edge C→E is included in Graph 1 and 2, not included in Graph 3, hence the edge feature of C→E is [1,1,0].


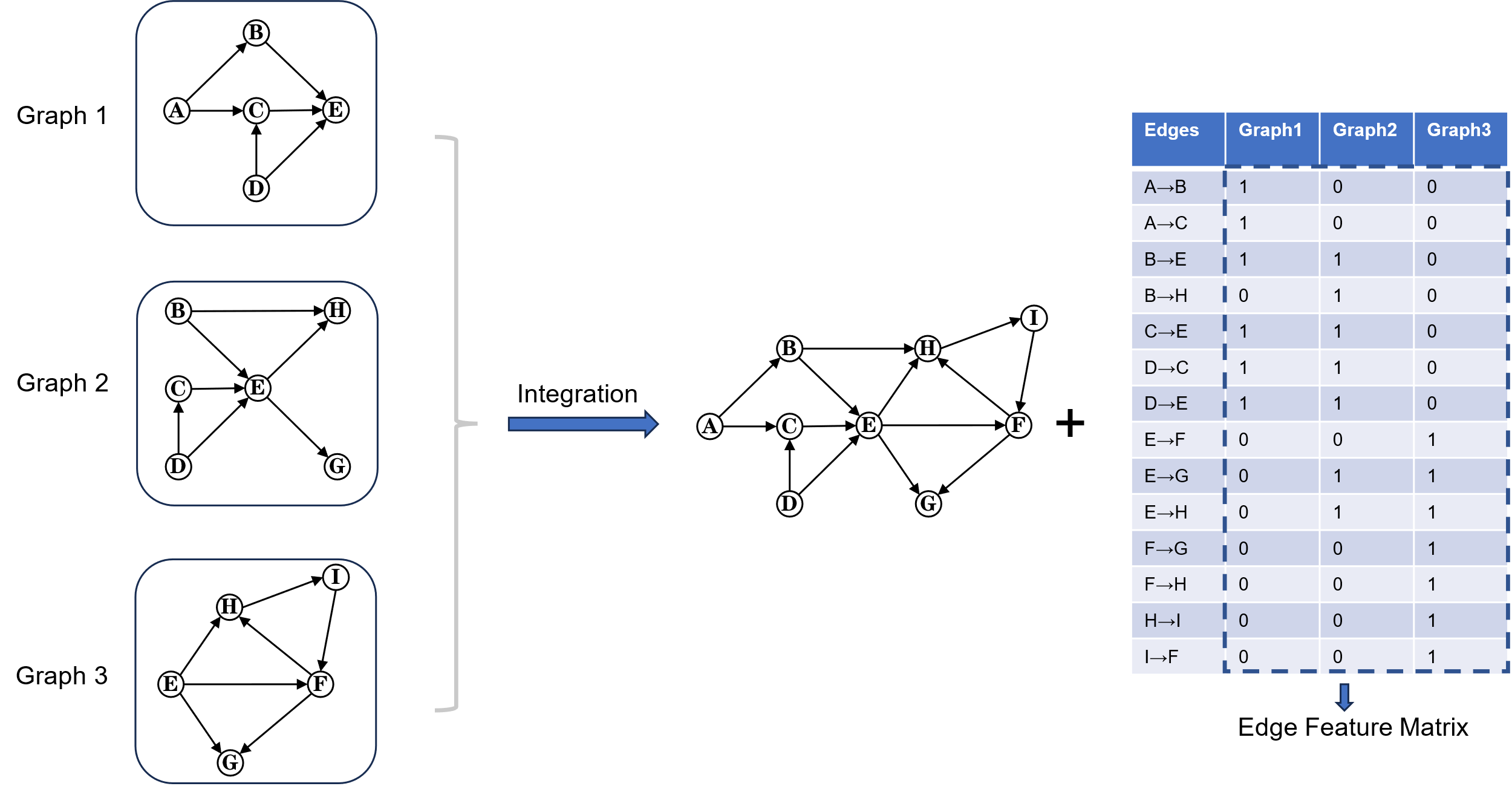


Figure S10: **Schematic of how DGP-AMIO integrates gene interaction networks from different databases.**

### 2.3 Hyper-Parameter Optimization

The DGP-AMIO contains several hyperparameters, including the dimension of the hidden node features, the number of attention heads, dropout rate, learning rate and weight decay, which influence the performance of the model. Thus it is crucial to find the optimal combination of hyperparameters.

We initially divided the labeled samples into an 80% training set and a 20% test set. We performed a grid search on the training set to explore various hyperparameter combinations and chose the best one. Considering the limited number of labeled samples, we employed five-fold cross-validation to mitigate the effect of data splitting on model performance. During the five-fold cross-validation process, we divided the training set into five equal parts (see Figure S11). For each hyperparameter combination, we trained five models, with each model utilizing 80% of the training set for training. After training, we computed the Area Under the Precision-Recall Curve (AUPR) on the remaining 20% of the training data. In this process, each gene is used for training four times and once for validation. This procedure yielded five AUPR values for each hyperparameter combination. Finally, taking into account both model performance (mean/median) and robustness (variance), we selected the hyperparameter combination that exhibited the best overall performance.


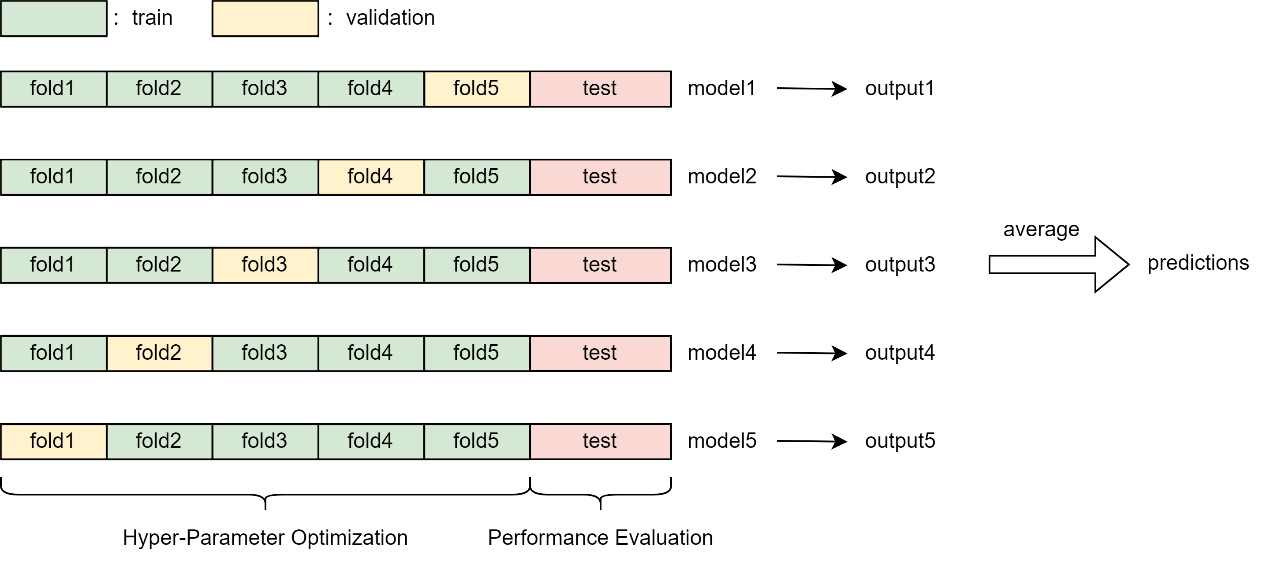


Figure S11: **Data Splitting.** The labeled data was divided into an 80% training set and a 20% test set. The training set was further divided into five equal parts for five-fold cross-validation, involving model training and hyperparameter optimization. The test set was used to evaluate the model performance. The average output of the five models obtained through cross-validation was considered as the final predictions.

### 2.4 Training and Performance Evaluation

We followed the data splitting approach described in the previous section, shown as Figure S11. During each data splitting, we ensure that the ratio of positive to negative samples remained consistent. During the training phase, we employed five-fold cross validation on the training set to train five models. During the testing phase, we utilize two evaluation methods: 1. Individual Model Evaluation: Each of the five models is tested on the test set, and five sets of AUROC and AUPR values are computed. The performance and robustness of the model are assessed by calculating the mean and variance of these five metrics. 2. Ensemble-Based Evaluation: Inspired by ensemble learning, we calculated the average output of the five models and use this average to compute AUROC and AUPR on the test set.

When comparing DGP-AMIO with other methods, the same training and evaluation methods described above are employed for the other methods.

### 2.5 Prediction

After training and testing, DGP-AMIO gives each gene a probability of being disease genes between 0 and 1. As five-fold cross-validation was employed during the training phase, we consider the average output of the five models as the final prediction result (see Figure S11).

### 2.6 Other Methods for Performance Comparison

- **EMOGI(**[**Schulte-Sasse, et al., 2021**](#_ENREF_12)**):** A method based on graph convolutional networks for predicting pan-cancer genes. The input of EMOGI is an undirected graph constructed based on the protein-protein interaction (PPI) network, multiomics data as node features, and known pan-cancer genes as partially labeled samples. This framework, similar to DGP-AMIO, addresses the semi-supervised node binary classification problem using graph neural networks and can be easily adapted to other disease datasets. During performance comparison, we used the PPI network from STRING([Szklarczyk, et al., 2019](#_ENREF_13)) for graph construction and disease omics data as node features (consistent with DGP-AMIO and other methods).
- **CNNC(**[**Yuan and Bar-Joseph, 2019**](#_ENREF_16)**):** A CNN-based deep learning method for inferring gene relationships from gene expression data. CNNC first calculates the Normalized Empirical Probability Distribution Function (NEPDF) for each gene pair based on their expression data. The 2D NEPDF images are then used as inputs of a convolutional neural network (CNN) to predict the relationship type between gene pairs. CNNC is a general framework for inferring gene-gene relationships and disease gene prediction is one of its tasks. When training CNNC for disease gene prediction, the input is NEPDF of positive pairs (disease gene-disease gene) and negative pairs (disease gene-unknown gene). When testing and predicting, the gene to predict is paired with all disease genes in the training set and CNNC computes the average of the outputs of these gene pairs as the probability of being a disease gene.
- **Node2vec+Random Forest:** A machine learning method that can incorporate both graph structure information and omics. Firstly a graph is constructed based on the gene interaction network, and node2vec is utilized to obtain low-dimensional representations of the nodes in the graph. Node2vec([Grover and Leskovec, 2016](#_ENREF_1)) is an algorithm that learns vector representations of nodes in a graph based on random walks. It can extract both local and global structure information by balancing between breadth-first search and depth-first search strategies, improving based on DeepWalk([Perozzi, et al., 2014](#_ENREF_9)). Then the node embeddings learned by node2vec are concatenated with omics data to generate the gene feature matrix. Finally the gene features and labels are used to train a random forest binary classifier.

### 2.7 Omics Data

We used three types of omics data: gene expression, DNA methylation, and gene mutations, as initial node features. For non-cancer diseases, we only utilized gene expression data obtained from the Gene Expression Omnibus (GEO) database, because it is hard to obtain the DNA methylation and gene mutation data of non-cancer diseases. In the case of cancer diseases, in addition to GEO gene expression data, we also utilized DNA methylation and gene mutation data obtained from The Cancer Genome Atlas (TCGA) database.

The Table S4 provided below lists the GEO accession numbers corresponding to the gene expression data we used. Users can retrieve and download the data by searching for these accession numbers on the GEO website. Alternatively, users may also choose to utilize their own omics data.

Table S4: **GEO accession numbers of gene expression data we used to train DGP-AMIO.**

| Disease | GEO Accession | Disease | GEO Accession |
| --- | --- | --- | --- |
| Asthma | GSE143303 | psoriasis | GSE66511 |
| Alzheimer | GSE184942 | Rheumatoid arthritis |  |
| Idiopathic pulmonary fibrosis (IPF) | GSE185492 | Prostate cancer | GSE114740 |
| Myeloma multiple | GSE167968 | Malignant melanoma | GSE140673 |
| Inflammatory bowel disease | GSE227747 | Lung adenocarcinoma | GSE166720 |
| Triple-negative breast cancer (TNBC) | GSE181466 | Glioblastoma multiforme | GSE179649 |
| Hepatocellular carcinoma | GSE202853 | Gastrointestinal Stromal Tumors (GIST) | GSE136755 |
| diffuse large B-cell lymphoma (DLBCL) | GSE178965 | Gastric cancer | GSE152415 |
| Sclerosis | GSE130955 | Colorectal cancer | GSE192667 |
| Cervical cancer | GSE49288 | Breast cancer | GSE196723 |

### 2.8 Disease Genes

#### 2.8.1 Disease Genes for Training and Testing

Known disease genes of 20 diseases were collected from Malacards (<https://www.malacards.org/>). For each disease, these genes (positive samples) and an equal number of randomly sampled unknown genes (negative samples) form the labeled dataset, which is split and used for training and test. Table S5 lists the number of disease genes for each disease on Malacards, as well as the number of disease genes actually used for training and testing after data overlap (the removal of disease genes that are not present in the gene interaction networks or lack omics).

Table S5: **The number of disease genes on Malacards** ($n_{raw}$) **and the number of** **disease genes used for training and testing after data overlap** ($n_{overlap}$)

| Disease | $n_{raw}$ | $n_{overlap}$ | Disease | $n_{raw}$ | $n_{overlap}$ |
| --- | --- | --- | --- | --- | --- |
| Asthma | 306 | 291 | psoriasis | 191 | 153 |
| Alzheimer | 470 | 403 | Rheumatoid arthritis | 261 | 246 |
| Idiopathic pulmonary fibrosis (IPF) | 172 | 153 | Prostate cancer | 709 | 577 |
| Myeloma multiple | 225 | 207 | Malignant melanoma | 404 | 349 |
| Inflammatory bowel disease | 393 | 369 | Lung adenocarcinoma | 876 | 593 |
| Triple-negative breast cancer | 598 | 571 | Glioblastoma multiforme | 267 | 231 |
| Hepatocellular carcinoma | 676 | 435 | Gastrointestinal Stromal Tumors (GIST) | 101 | 96 |
| diffuse large B-cell lymphoma (DLBCL) | 97 | 69 | Gastric cancer | 516 | 392 |
| Sclerosis | 204 | 175 | Colorectal cancer | 1074 | 972 |
| Cervical cancer | 150 | 87 | Breast cancer | 1056 | 888 |

#### 2.8.2 Independent Disease Gene Sets

We collected disease genes not included in Malacards from other sources to construct independent test sets for evaluating DGP-AMIO’s ability of predicting novel disease genes and performance comparison. The independent disease genes are collected from:

- DisGeNet([Piñero, et al., 2017](#_ENREF_10)): a database of gene-disease associations. (<https://www.disgenet.org/>)
- AllerGAtlas([Liu, et al., 2018](#_ENREF_6)): a human allergy-related genes database, where we collected asthma-related genes. (<http://biokb.ncpsb.org.cn/AllerGAtlas/>)

### 2.9 Gene Interaction Networks

The gene interaction networks used in this work are listed as below. We also provide the website addresses where the data can be downloaded.

- KEGG([Kanehisa and Goto, 2000](#_ENREF_4)) (Kyoto Encyclopedia of Genes and Genomes) pathways: a collection of knowledge including molecular interactions, biological pathways, and functional annotations of genes and proteins. KEGGREST (a package in R) can be used to download totally 345 KEGG pathways of human, and KEGGgraph (a package in R) provide the function parseKGML2Graph to convert each pathway into a directed graph and function mergeGraphs to merge all graphs into a single graph.
- RegNetwork([Liu, et al., 2015](#_ENREF_7)): a database of transcriptional and post-transcriptional regulatory relationships in human and mouse. (<https://regnetworkweb.org/>download.jsp)
- TRRUST([Han, et al., 2015](#_ENREF_2)): a manually curated database of human and mouse transcriptional regulatory networks. (<https://www.grnpedia.org/trrust/>downloadnetwork.php)
- EVEX([Van Landeghem, et al., 2012](#_ENREF_14)): a text mining resource built on top of PubMed abstracts and PubMed Central full texts, containing association between genes and proteins. (<http://evexdb.org/download/network-format/Metazoa/>)
- JASPAR([Mathelier, et al., 2014](#_ENREF_8)): a database of transcription factor binding profiles.

(<https://maayanlab.cloud/Harmonizome/dataset/JASPAR+Predicted+Transcription+Factor+Targets>)

- CHEA([Lachmann, et al., 2010](#_ENREF_5)): a database of transcription factor regulations inferred from integrating genome-wide ChIP-X experiments.

(<https://maayanlab.cloud/Harmonizome/dataset/CHEA+Transcription+Factor+Targets>)

- MOTIFMAP([Xie, et al., 2009](#_ENREF_15)): a database of target genes of transcription factors predicted using known transcription factor binding site motifs.

(<https://maayanlab.cloud/Harmonizome/dataset/MotifMap+Predicted+Transcription+Factor+Targets>)

- STRING([Szklarczyk, et al., 2019](#_ENREF_13)): a database containing PPI (protein-protein interaction) networks of different organisms.

(<https://string-db.org/cgi/download?sessionId=bDYbafVQ6nqL&species_text=Homo+sapiens>)

- CPDB([Kamburov, et al., 2013](#_ENREF_3)) (The ConsensusPathDB): a molecular functional interaction [database](https://en.wikipedia.org/wiki/Database), containing [protein and gene interactions](https://en.wikipedia.org/wiki/Protein_interaction) in humans. (<http://cpdb.molgen.mpg.de/>)
- IRefIndex([Razick, et al., 2008](#_ENREF_11)): a protein interaction database.

(<https://irefindex.vib.be/wiki/index.php/README_MITAB2.6_for_iRefIndex_20.0>)

We integrated gene interaction networks from 10 databases: KEGG, RegNetwork, TRRUST, EVEX, JASPAR, CHEA, MOTIFMAP, STRING, CPDB and IRefIndex. Table S6 lists the number of genes and interactions in different gene interaction networks. Note that these results are after data preprocessing, and the preprocessing methods can be found in the main "Methods" section.

Table S6: **The number of genes (nodes) and interactions (directed edges) in different gene interaction networks.**

| Gene Interaction Networks | Number of Genes (nodes) | Number of interactions (directed edges) |
| --- | --- | --- |
| KEGG | 6314 | 62972 |
| RegNetwork | 15512 | 211482 |
| TRRUST | 2861 | 8403 |
| EVEX | 13247 | 236165 |
| JASPAR | 21545 | 148052 |
| CHEA | 21584 | 386696 |
| MOTIFMAP | 20686 | 158849 |
| STRING | 12411 | 279008 |
| CPDB | 14178 | 664824 |
| IRefIndex | 17757 | 917272 |
| Integrate all | 27513 | 2426460 |

## References

Grover, A. and Leskovec, J. node2vec: Scalable feature learning for networks. In, *Proceedings of the 22nd ACM SIGKDD international conference on Knowledge discovery and data mining*. 2016. p. 855-864.

Han, H.*, et al.* TRRUST: a reference database of human transcriptional regulatory interactions. *Scientific reports* 2015;5(1):11432.

Kamburov, A.*, et al.* The ConsensusPathDB interaction database: 2013 update. *Nucleic acids research* 2013;41(D1):D793-D800.

Kanehisa, M. and Goto, S. KEGG: kyoto encyclopedia of genes and genomes. *Nucleic acids research* 2000;28(1):27-30.

Lachmann, A.*, et al.* ChEA: transcription factor regulation inferred from integrating genome-wide ChIP-X experiments. *Bioinformatics* 2010;26(19):2438-2444.

Liu, J.*, et al.* AllerGAtlas 1.0: a human allergy-related genes database. *Database* 2018;2018:bay010.

Liu, Z.-P.*, et al.* RegNetwork: an integrated database of transcriptional and post-transcriptional regulatory networks in human and mouse. *Database* 2015;2015:bav095.

Mathelier, A.*, et al.* JASPAR 2014: an extensively expanded and updated open-access database of transcription factor binding profiles. *Nucleic acids research* 2014;42(D1):D142-D147.

Perozzi, B., Al-Rfou, R. and Skiena, S. Deepwalk: Online learning of social representations. In, *Proceedings of the 20th ACM SIGKDD international conference on Knowledge discovery and data mining*. 2014. p. 701-710.

Piñero, J.*, et al.* DisGeNET: a comprehensive platform integrating information on human disease-associated genes and variants. *Nucleic Acids Research* 2017;45(D1):D833-D839.

Razick, S., Magklaras, G. and Donaldson, I.M. iRefIndex: a consolidated protein interaction database with provenance. *BMC bioinformatics* 2008;9(1):1-19.

Schulte-Sasse, R.*, et al.* Integration of multiomics data with graph convolutional networks to identify new cancer genes and their associated molecular mechanisms. *Nat Mach Intell* 2021;3(6):513-526.

Szklarczyk, D.*, et al.* STRING v11: protein–protein association networks with increased coverage, supporting functional discovery in genome-wide experimental datasets. *Nucleic acids research* 2019;47(D1):D607-D613.

Van Landeghem, S.*, et al.* Exploring biomolecular literature with EVEX: connecting genes through events, homology, and indirect associations. *Advances in bioinformatics* 2012;2012.

Xie, X., Rigor, P. and Baldi, P. MotifMap: a human genome-wide map of candidate regulatory motif sites. *Bioinformatics* 2009;25(2):167-174.

Yuan, Y. and Bar-Joseph, Z. Deep learning for inferring gene relationships from single-cell expression data. *Proceedings of the National Academy of Sciences* 2019;116(52):27151-27158.
